# Supplementary material for: Low-dose aspirin is not effective as an adjunct treatment for HIV infection among people living with HIV on dolutegravir-based antiretroviral therapy: A randomised double-blind, parallel-group placebo-controlled trial
Source: PLoS One. 2025 Aug 29;20(8):e0331087. doi: 10.1371/journal.pone.0331087 (PMC12396663; doi:10.1371/journal.pone.0331087)
Supplement: S1 Table — (DOCX) [file pone.0331087.s002.docx]

**S1 Table. Logistic regression model using generalised estimating equations on the effects of the treatment arm on virologic response.**

| **Variable** | | **Number** | **Odds ratio** | **95% Confidence Interval** | **p – value** |
| --- | --- | --- | --- | --- | --- |
| **Virologic suppression (< 50 RNA copies/mL)** | |  |  |  |  |
|  | Treatment (placebo arm vs aspirin arm) |  | 0.89 | 0.46 – 1.69 | 0.71 |
|  | Time |  |  |  |  |
|  | Baseline | 174 vs 172 | Reference |  |  |
|  | 24 weeks | 125 vs 106 | 23.30 | 12.40 – 43.76 | 0.000 |
|  | Treatment x time |  |  |  |  |
|  | Treatment x baseline |  | Reference |  |  |
|  | Treatment x 24 weeks |  | 1.32 | 0.55 – 3.16 | 0.53 |
| **Virologic failure (≥ 1000 RNA copies/mL)** | |  |  |  |  |
|  | Treatment (placebo arm vs aspirin arm) |  | 1.01 | 0.57 – 1.80 | 0.96 |
|  | Time |  |  |  |  |
|  | Baseline | 174 vs 172 | Reference |  |  |
|  | 24 weeks | 125 vs 106 | 0.01 | 0.003 – 0.02 | 0.000 |
|  | Treatment x time |  |  |  |  |
|  | Treatment x baseline |  | Reference |  |  |
|  | Treatment x 24 weeks |  | 1.05 | 0.25 – 4.40 | 0.95 |
