# Supplementary figures and images for: Low-dose aspirin is not effective as an adjunct treatment for HIV infection among people living with HIV on dolutegravir-based antiretroviral therapy: A randomised double-blind, parallel-group placebo-controlled trial
Source: PLoS One. 2025 Aug 29;20(8):e0331087. doi: 10.1371/journal.pone.0331087 (PMC12396663; doi:10.1371/journal.pone.0331087)

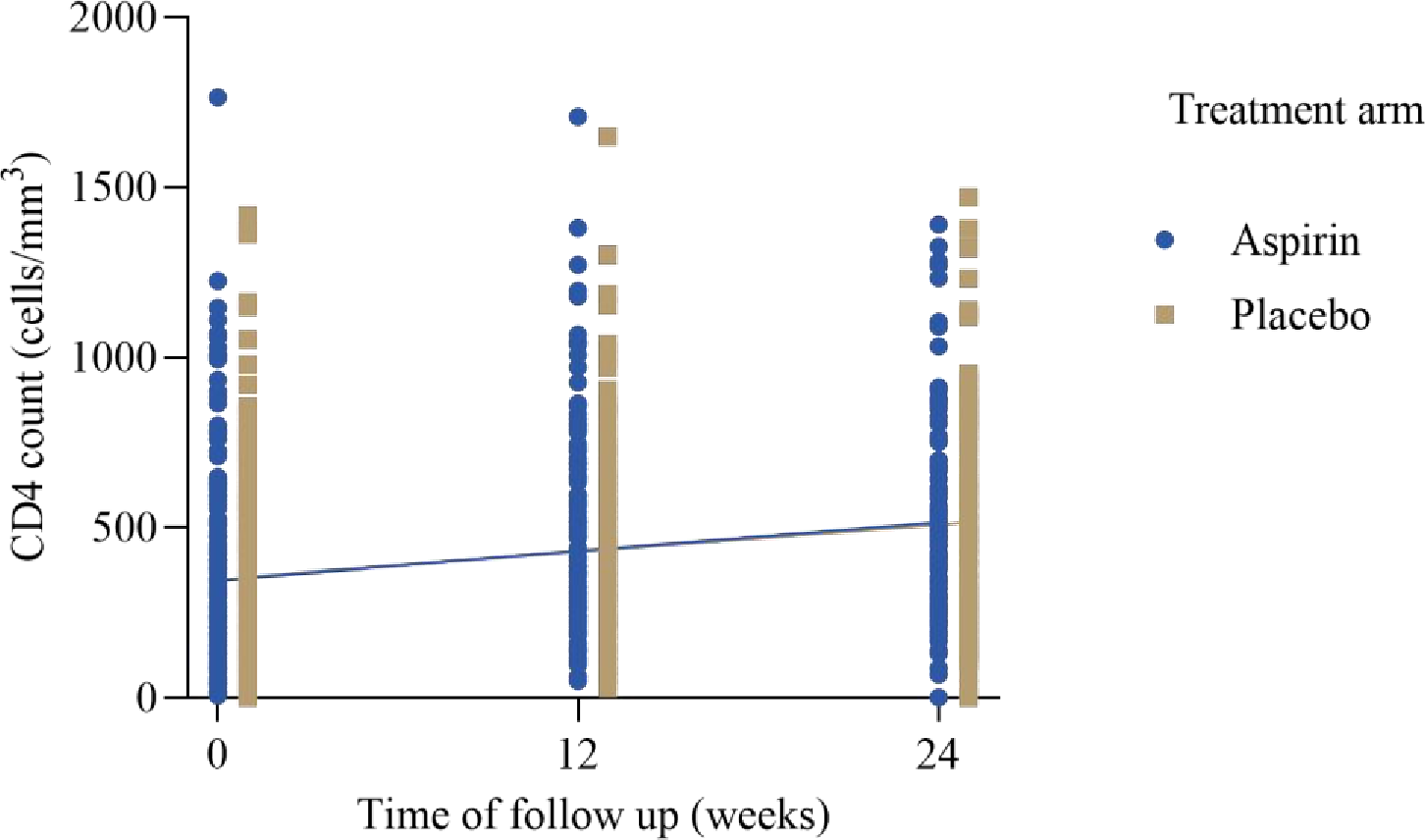

Supplement: S1 Fig — Note: to allow visualisation of all the values, the columns for the arms are slightly offset. (TIF) [file pone.0331087.s003.tif]

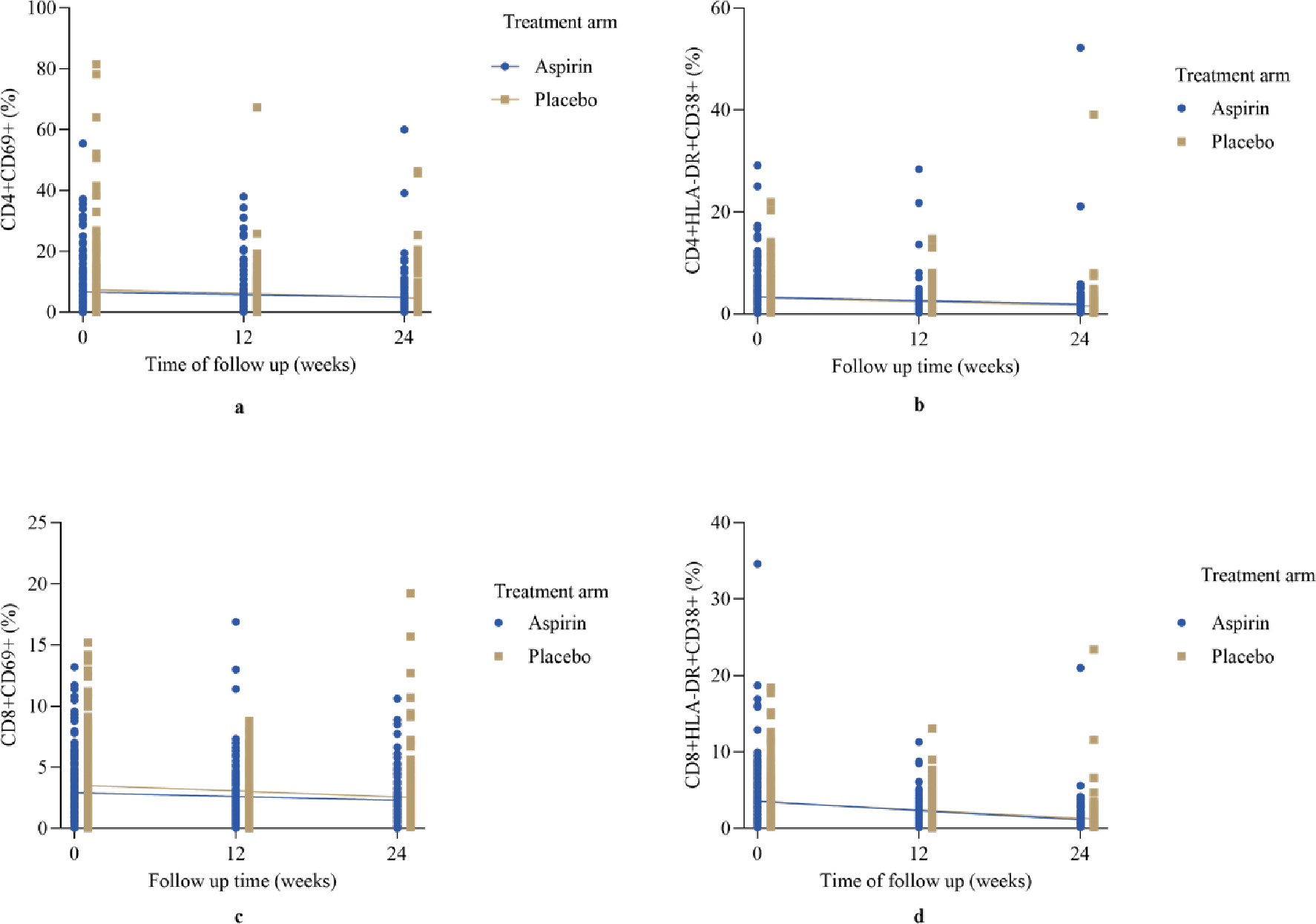

Supplement: S2 Fig — Changes in CD4+CD69+ % b. Changes in CD4+HLA-DR+CD38+ % c. Changes in CD8+CD69+ % d. Changes in CD8+HLA-DR+CD38+ %. Note: to allow visualisation of all the values, the columns for the arms are slightly offset. (TIF) [file pone.0331087.s004.tif]

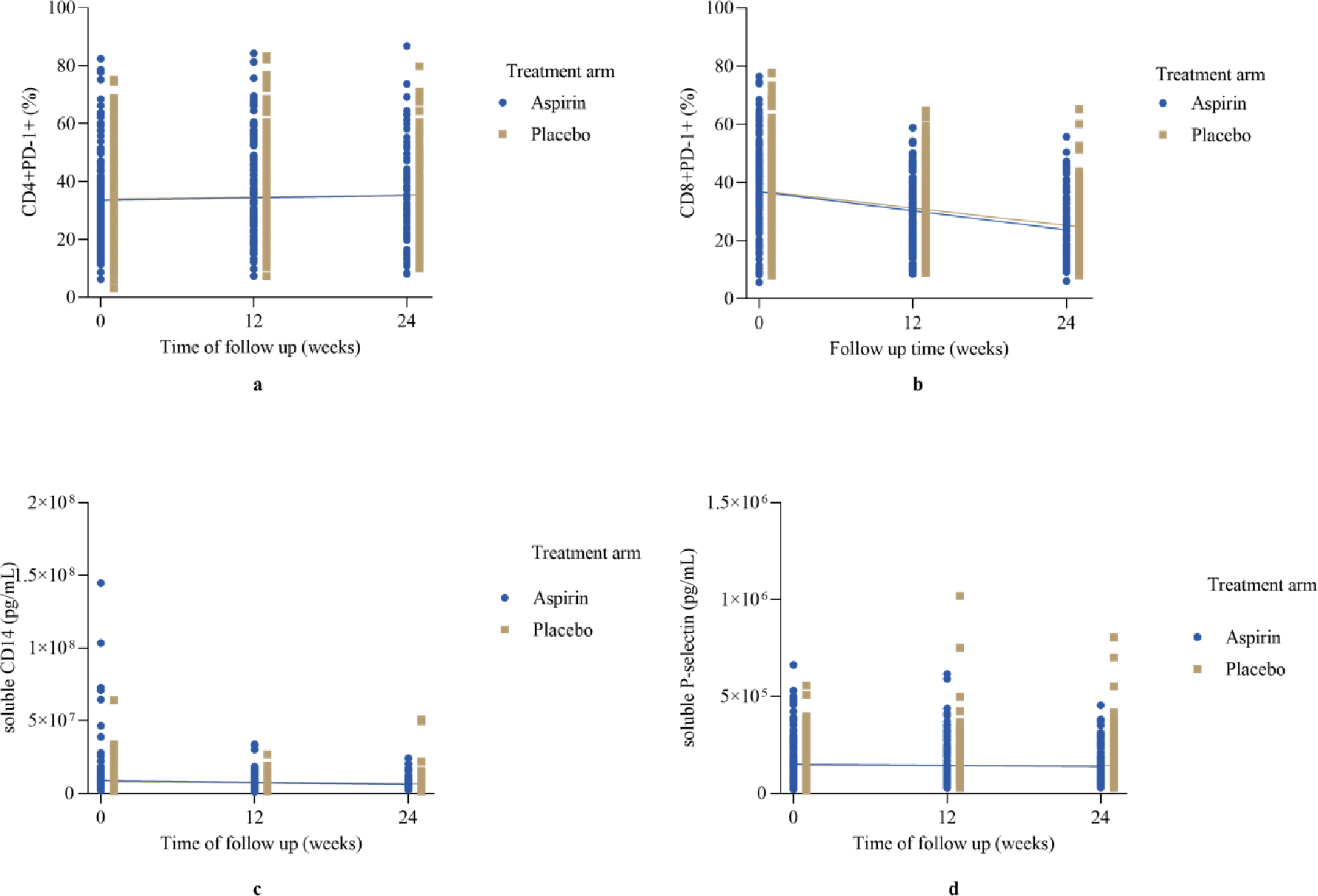

Supplement: S3 Fig — Changes in CD4+PD-1+ % b. Changes in CD8+PD-1+ % c. Changes in soluble CD14 (pg/mL) d. Changes in soluble P-selectin (pg/mL). Note: to allow visualisation of all the values, the columns for the arms are slightly offset. (TIF) [file pone.0331087.s005.tif]
