# Supplementary material for: Low-dose aspirin is not effective as an adjunct treatment for HIV infection among people living with HIV on dolutegravir-based antiretroviral therapy: A randomised double-blind, parallel-group placebo-controlled trial
Source: PLoS One. 2025 Aug 29;20(8):e0331087. doi: 10.1371/journal.pone.0331087 (PMC12396663; doi:10.1371/journal.pone.0331087)
Supplement: S2 Table — (DOCX) [file pone.0331087.s006.docx]

| **S2 Table. Linear regression model using generalised estimating equations on the effects of the treatment arm on CD4 count, platelet activation, immune activation and exhaustion markers.** | | | | | |
| --- | --- | --- | --- | --- | --- |
| **Variable** | | **Number** | **β** | **95% Confidence Interval** | **p – value** |
| **CD4 count (cells/mm^3^)** | |  |  |  |  |
|  | Treatment (placebo arm vs aspirin arm) |  | 8.80 | -47.92 – 65.53 | 0.76 |
|  | Time |  |  |  |  |
|  | Baseline | 210 vs 214 | Reference |  |  |
|  | 12 weeks | 149 vs 141 | 120.60 | 79.71 – 161.50 | 0.000 |
|  | 24 weeks | 130 vs 112 | 160.57 | 112.61 – 208.53 | 0.000 |
|  | Treatment x time |  |  |  |  |
|  | Treatment x baseline |  | Reference |  |  |
|  | Treatment x 12 weeks |  | -2.79 | -59.06 – 53.48 | 0.92 |
|  | Treatment x 24 weeks |  | -8.54 | -73.96 – 56.89 | 0.80 |
| **CD4+CD69+ (%)** | |  |  |  |  |
|  | Treatment (placebo arm vs aspirin arm) |  | 1.11 | -0.88 – 3.10 | 0.27 |
|  | Time |  |  |  |  |
|  | Baseline | 202 vs 204 | Reference |  |  |
|  | 12 weeks | 141 vs 132 | -1.02 | -2.52 – 0.48 | 0.18 |
|  | 24 weeks | 114 vs 101 | -1.62 | -3.45 – 0.21 | 0.08 |
|  | Treatment x time |  |  |  |  |
|  | Treatment x baseline |  | Reference |  |  |
|  | Treatment x 12 weeks |  | -1.91 | -4.20 – 0.37 | 0.10 |
|  | Treatment x 24 weeks |  | -0.72 | -3.48 – 2.04 | 0.61 |
| **CD4+PD-1+ (%)** | |  |  |  |  |
|  | Treatment (placebo arm vs aspirin arm) |  | 0.38 | -2.35 – 3.10 | 0.79 |
|  | Time |  |  |  |  |
|  | Baseline | 208 vs 212 | Reference |  |  |
|  | 12 weeks | 143 vs 134 | 3.57 | 1.13 – 6.02 | 0.004 |
|  | 24 weeks | 117 vs 102 | 1.07 | -1.79 – 3.93 | 0.46 |
|  | Treatment x time |  |  |  |  |
|  | Treatment x baseline |  | Reference |  |  |
|  | Treatment x 12 weeks |  | 0.38 | -3.12 – 3.87 | 0.83 |
|  | Treatment x 24 weeks |  | -0.43 | -4.23 – 3.36 | 0.82 |
| **CD4+HLA-DR+CD38+ (%)** | |  |  |  |  |
|  | Treatment (placebo arm vs aspirin arm) |  | -0.31 | -1.05 – 0.42 | 0.40 |
|  | Time |  |  |  |  |
|  | Baseline | 203 vs 206 | Reference |  |  |
|  | 12 weeks | 143 vs 133 | -1.12 | -1.82 – -0.43 | 0.002 |
|  | 24 weeks | 116 vs 102 | -1.31 | -2.51 – -0.12 | 0.03 |
|  | Treatment x time |  |  |  |  |
|  | Treatment x baseline |  | Reference |  |  |
|  | Treatment x 12 weeks |  | 0.17 | -0.70 – 1.04 | 0.86 |
|  | Treatment x 24 weeks |  | -0.12 | -1.49 – 1.24 | 0.82 |
| **CD8+CD69+ (%)** | |  |  |  |  |
|  | Treatment (placebo arm vs aspirin arm) |  | 0.72 | 0.18 – 1.27 | 0.01 |
|  | Time |  |  |  |  |
|  | Baseline | 202 vs 209 | Reference |  |  |
|  | 12 weeks | 143 vs 133 | -0.48 | -0.95 – -0.01 | 0.04 |
|  | 24 weeks | 115 vs 100 | -0.58 | -1.03 – -0.14 | 0.01 |
|  | Treatment x time |  |  |  |  |
|  | Treatment x baseline |  | Reference |  |  |
|  | Treatment x 12 weeks |  | -0.68 | -1.34 – -0.02 | 0.04 |
|  | Treatment x 24 weeks |  | -0.25 | -1.04 – 0.40 | 0.53 |
| **CD8+PD-1+ (%)** | |  |  |  |  |
|  | Treatment (placebo arm vs aspirin arm) |  | 0.11 | -2.81 – 3.04 | 0.94 |
|  | Time |  |  |  |  |
|  | Baseline | 208 vs 212 | Reference |  |  |
|  | 12 weeks | 143 vs 134 | -8.56 | -10.67 – -6.46 | 0.000 |
|  | 24 weeks | 117 vs 102 | -12.57 | -14.83 – -10.31 | 0.000 |
|  | Treatment x time |  |  |  |  |
|  | Treatment x baseline |  | Reference |  |  |
|  | Treatment x 12 weeks |  | -0.35 | -1.91 – 4.35 | 0.44 |
|  | Treatment x 24 weeks |  | 1.22 | -1.04 – 0.40 | 0.53 |
| **CD8+HLA-DR+CD38+ (%)** | |  |  |  |  |
|  | Treatment (placebo arm vs aspirin arm) |  | -0.16 | -0.83 – 0.51 | 0.64 |
|  | Time |  |  |  |  |
|  | Baseline | 207 vs 209 | Reference |  |  |
|  | 12 weeks | 143 vs 134 | -1.97 | -2.47 – -1.47 | 0.000 |
|  | 24 weeks | 116 vs 102 | -2.24 | -2.84 – -1.64 | 0.000 |
|  | Treatment x time |  |  |  |  |
|  | Treatment x baseline |  | Reference |  |  |
|  | Treatment x 12 weeks |  | 0.35 | -0.36 – 1.05 | 0.34 |
|  | Treatment x 24 weeks |  | 0.15 | -0.67 – 0.96 | 0.73 |
| **Soluble CD14 (pg/nL)** | |  |  |  |  |
|  | Treatment (placebo arm vs aspirin arm) |  | -0.91 | -3.10 – 1.28 | 0.42 |
|  | Time |  |  |  |  |
|  | Baseline | 211 vs 213 | Reference |  |  |
|  | 12 weeks | 149 vs 137 | -1.98 | -3.86 – -0.10 | 0.04 |
|  | 24 weeks | 124 vs 105 | -2.55 | -4.64 – -0.45 | 0.02 |
|  | Treatment x time |  |  |  |  |
|  | Treatment x baseline |  | Reference |  |  |
|  | Treatment x 12 weeks |  | 0.65 | -1.50 – 2.79 | 0.28 |
|  | Treatment x 24 weeks |  | 1.34 | -1.08 – 3.76 | 0.73 |
| **Soluble P-selectin (pg/nL)** | |  |  |  |  |
|  | Treatment (placebo arm vs aspirin arm) |  | -0.01 | -0.03 – 0.02 | 0.62 |
|  | Time |  |  |  |  |
|  | Baseline | 211 vs 213 | Reference |  |  |
|  | 12 weeks | 149 vs 138 | 0.003 | -0.02 – -0.03 | 0.77 |
|  | 24 weeks | 123 vs 105 | -0.01 | -0.04 – -0.01 | 0.22 |
|  | Treatment x time |  |  |  |  |
|  | Treatment x baseline |  | Reference |  |  |
|  | Treatment x 12 weeks |  | 0.004 | -0.03 – 0.04 | 0.80 |
|  | Treatment x 24 weeks |  | 0.01 | -0.02 – 0.04 | 0.49 |
