# Supplementary material for: Low-dose aspirin is not effective as an adjunct treatment for HIV infection among people living with HIV on dolutegravir-based antiretroviral therapy: A randomised double-blind, parallel-group placebo-controlled trial
Source: PLoS One. 2025 Aug 29;20(8):e0331087. doi: 10.1371/journal.pone.0331087 (PMC12396663; doi:10.1371/journal.pone.0331087)
Supplement: S3 Table — (DOCX) [file pone.0331087.s007.docx]

| **Laboratory test** | **Aspirin arm** | **Placebo arm** | **p** |
| --- | --- | --- | --- |
| Platelet count (x 10^3^ cell/µL) median (IQR), (109 aspirin arm, 123 placebo arm) | -15 (-73, 35) | -21.0 (-87, 22) | 0.24 |
| Haemoglobin concentration (g/dL) median (IQR) (109 aspirin arm, 123 placebo arm) | -0.3 (-1.0, 0.8) | -0.4 (-1.5, 0.9) | 0.26 |
| Aspartate transferase level (IU/L) median (IQR) (19 aspirin arm, 20 placebo arm) | 0.5 (-8.7, 12.3) | -2.4 (-9.2, -17.5) | 1.000 |
| Alanine transferase level (IU/L) median (IQR) (19 aspirin arm, 20 placebo arm) | -3.4 (-10.7, 1.8) | -5.4 (-10.0, 2.5) | 0.71 |
| eGFR (mL/min/1.73m^2^) median (IQR), (53 aspirin arm, 56 placebo arm) | 8.72 (-9.87, 35.61) | 13.04 (-8.95, 34.65) | 0.80 |
| Abbreviations: IQR= interquartile range; eGFR= Estimated glomerular filtration rate  Notes: p-values are based on Mann-Whitney U tests | | | |

**S3 Table. Laboratory values median changes from baseline among participants at week 24.**
