# Supplementary material for: Low-dose aspirin is not effective as an adjunct treatment for HIV infection among people living with HIV on dolutegravir-based antiretroviral therapy: A randomised double-blind, parallel-group placebo-controlled trial
Source: PLoS One. 2025 Aug 29;20(8):e0331087. doi: 10.1371/journal.pone.0331087 (PMC12396663; doi:10.1371/journal.pone.0331087)
Supplement: S4 Table — Notes: p-value based on Fisher exact test; thrombocytopenia = platelet count < 150 x 103 cell/µL. (DOCX) [file pone.0331087.s008.docx]

|  |  |  | **Week 24** | |  |  |
| --- | --- | --- | --- | --- | --- | --- |
| **Arm** | **Baseline** | | Normal platelet count | Thrombocytopenia | **Total** | **P - value** |
| Aspirin arm |  | Normal platelet count | 88 (94.6) | 5 (5.4) | 93 (100) | 1.00 |
|  |  | Thrombocytopenia | 12 (75.0) | 4 (25.0) | 16 (100) |  |
|  |  |  |  |  |  |  |
| Placebo arm |  | Normal platelet count | 89 (94.7) | 5 (5.3) | 94 (100) |  |
|  |  | Thrombocytopenia | 20 (69.0) | 9 (31.0) | 29 (100) |  |

**S4 Table. Proportion of thrombocytopenia at week 24.**
