# Supplementary material for: Low-dose aspirin is not effective as an adjunct treatment for HIV infection among people living with HIV on dolutegravir-based antiretroviral therapy: A randomised double-blind, parallel-group placebo-controlled trial
Source: PLoS One. 2025 Aug 29;20(8):e0331087. doi: 10.1371/journal.pone.0331087 (PMC12396663; doi:10.1371/journal.pone.0331087)
Supplement: S5 Table — Notes: p-value based on Fisher exact test; anaemia = Haemoglobin concentration < 12.0 g/dl for females and Haemoglobin concentration < 13.0 g/dl for males. (DOCX) [file pone.0331087.s009.docx]

|  |  |  | **Week 24** | |  |  |
| --- | --- | --- | --- | --- | --- | --- |
| **Arm** | **Baseline** | | Normal haemoglobin concentration | Anaemia | **Total** | **P - value** |
| Aspirin arm |  | Normal haemoglobin concentration | 32 (76.2) | 10 (23.8) | 42 (100) | 1.00 |
|  |  | Anaemia | 14 (20.9) | 53 (79.1) | 67 (100) |  |
|  |  |  |  |  |  |  |
| Placebo arm |  | Normal haemoglobin concentration | 34 (77.3) | 10 (22.7) | 44 (100) |  |
|  |  | Anaemia | 23 (29.1) | 56 (70.9) | 79 (100) |  |

**S5 Table. Proportion of anaemia at week 24.**
