# Supplementary material for: Low-dose aspirin is not effective as an adjunct treatment for HIV infection among people living with HIV on dolutegravir-based antiretroviral therapy: A randomised double-blind, parallel-group placebo-controlled trial
Source: PLoS One. 2025 Aug 29;20(8):e0331087. doi: 10.1371/journal.pone.0331087 (PMC12396663; doi:10.1371/journal.pone.0331087)
Supplement: S6 Table — Notes: p-value based on Fisher exact test; elevated ALT levels = ALT level ≥ 1.25 x 34 IU/L for females and ALT level ≥ 1.25 x 45 IU/L for males. (DOCX) [file pone.0331087.s010.docx]

|  |  |  | **Week 24** | |  |  |
| --- | --- | --- | --- | --- | --- | --- |
| **Arm** | **Baseline** | | Normal ALT levels | Elevated ALT levels | **Total** | **P - value** |
| Aspirin arm |  | Normal ALT levels | 19 (100) | 0 (0.00) | 19 (100) | 1.00 |
|  |  | Elevated ALT levels | 0 (0.00) | 0 (0.00) | 0 (0.00) |  |
|  |  |  |  |  |  |  |
| Placebo arm |  | Normal ALT levels | 18 (94.7) | 1 (5.3) | 19 (100) |  |
|  |  | Elevated ALT levels | 1 (100) | 0 (0.00) | 1 (100) |  |

**S6 Table. Proportion of elevated ALT levels at week 24.**
