# Supplementary material for: Low-dose aspirin is not effective as an adjunct treatment for HIV infection among people living with HIV on dolutegravir-based antiretroviral therapy: A randomised double-blind, parallel-group placebo-controlled trial
Source: PLoS One. 2025 Aug 29;20(8):e0331087. doi: 10.1371/journal.pone.0331087 (PMC12396663; doi:10.1371/journal.pone.0331087)
Supplement: S7 Table — Notes: p-value based on Fisher exact test; elevated AST levels = AST level ≥ 1.25 x 31 IU/L for females and AST level ≥ 1.25 x 35 IU/L for males. (DOCX) [file pone.0331087.s011.docx]

|  |  |  | **Week 24** | |  |  |
| --- | --- | --- | --- | --- | --- | --- |
| **Arm** | **Baseline** | | Normal AST levels | Elevated AST levels | **Total** | **P - value** |
| Aspirin arm |  | Normal AST levels | 14 (87.5) | 2 (12.5) | 16 (100) | 1.00 |
|  |  | Elevated AST levels | 3 (100) | 0 (0.00) | 3 (100) |  |
|  |  |  |  |  |  |  |
| Placebo arm |  | Normal AST levels | 11 (84.6) | 2 (15.4) | 13 (100) |  |
|  |  | Elevated AST levels | 4 (57.1) | 3 (42.9) | 7 (100) |  |

**S7 Table. Proportion of elevated AST levels at week 24.**
