# Supplementary material for: Low-dose aspirin is not effective as an adjunct treatment for HIV infection among people living with HIV on dolutegravir-based antiretroviral therapy: A randomised double-blind, parallel-group placebo-controlled trial
Source: PLoS One. 2025 Aug 29;20(8):e0331087. doi: 10.1371/journal.pone.0331087 (PMC12396663; doi:10.1371/journal.pone.0331087)
Supplement: S8 Table — Abbreviation: CKD = chronic kidney disease; eGFR = estimated glomerular filtration rate Notes: p-value based on Fisher exact test; CKD = eGFR < 90 mL/min/1.73m2. (DOCX) [file pone.0331087.s012.docx]

|  |  |  | **Week 24** | |  |  |
| --- | --- | --- | --- | --- | --- | --- |
| **Arm** | **Baseline** | | Normal eGFR | CKD | **Total** | **P - value** |
| Aspirin arm |  | Normal eGFR | 25 (65.8) | 13 (34.2) | 38 (100) | 1.00 |
|  |  | CKD | 6 (40.0) | 9 (60.0) | 15 (100) |  |
|  |  |  |  |  |  |  |
| Placebo arm |  | Normal eGFR | 29 (67.4) | 14 (32.6) | 43 (100) |  |
|  |  | CKD | 5 (38.5) | 8 (61.5) | 13 (100) |  |

**S8 Table. Proportion of chronic kidney disease at week 24.**
