# Supplementary material for: Low-dose aspirin is not effective as an adjunct treatment for HIV infection among people living with HIV on dolutegravir-based antiretroviral therapy: A randomised double-blind, parallel-group placebo-controlled trial
Source: PLoS One. 2025 Aug 29;20(8):e0331087. doi: 10.1371/journal.pone.0331087 (PMC12396663; doi:10.1371/journal.pone.0331087)
Supplement: S2 File — (DOC) [file pone.0331087.s013.doc]

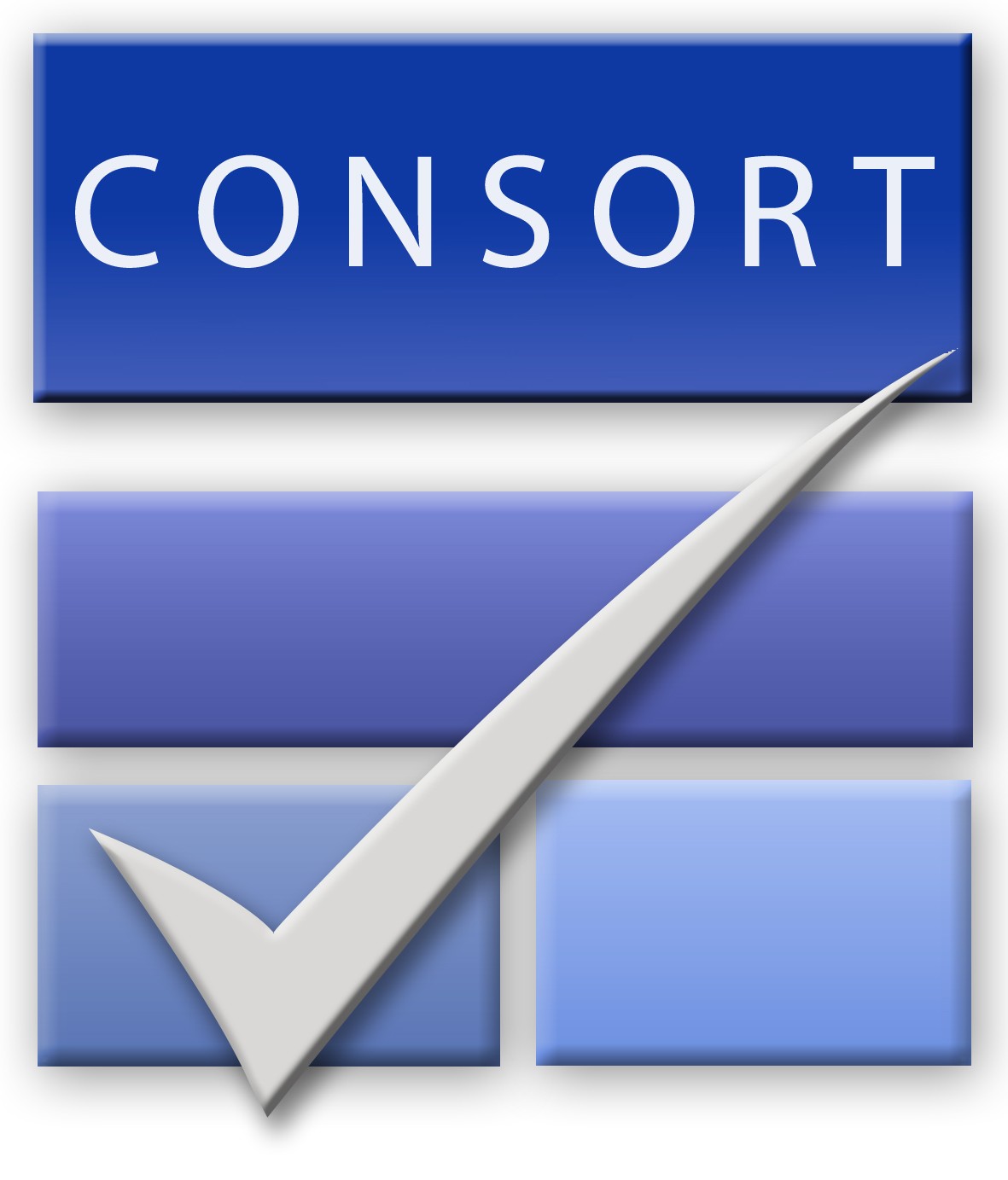
S2 File. CONSORT 2010 checklist of information to include when reporting a randomised trial*

| Section/Topic | Item No | Checklist item | Reported on page No |
| --- | --- | --- | --- |
| Title and abstract | | | |
|  | 1a | Identification as a randomised trial in the title | Page 1, Lines 2-3 |
|  | 1b | Structured summary of trial design, methods, results, and conclusions (for specific guidance see CONSORT for abstracts) | Page 2, Lines 25- Page 3, Lines 48 |
| Introduction | | | |
| Background and objectives | 2a | Scientific background and explanation of rationale | Page 5, Lines 94- Page 7 Lines 148 |
|  | 2b | Specific objectives or hypotheses | Page 7, Lines 149-153 |
| Methods | | | |
| Trial design | 3a | Description of trial design (such as parallel, factorial) including allocation ratio | Page 7, Line 156 & Page 8 Lines 172-173 |
|  | 3b | Important changes to methods after trial commencement (such as eligibility criteria), with reasons | N/A |
| Participants | 4a | Eligibility criteria for participants | Page 8, Lines 165-170 |
|  | 4b | Settings and locations where the data were collected | Page 7, Lines 156-163 |
| Interventions | 5 | The interventions for each group with sufficient details to allow replication, including how and when they were actually administered | Page 8, Lines 182- Page 9, Lines 202 |
| Outcomes | 6a | Completely defined pre-specified primary and secondary outcome measures, including how and when they were assessed | Page 12, Lines 264- Page 13, Lines 277 |
|  | 6b | Any changes to trial outcomes after the trial commenced, with reasons | Page 12, Lines 265-266 |
| Sample size | 7a | How sample size was determined | Page 13, Lines 279-282 |
|  | 7b | When applicable, explanation of any interim analyses and stopping guidelines | N/A |
| Randomisation: |  |  |  |
| Sequence generation | 8a | Method used to generate the random allocation sequence | Page 8, Lines 172-173 |
|  | 8b | Type of randomisation; details of any restriction (such as blocking and block size) | Page 8, Line 173 |
| Allocation concealment mechanism | 9 | Mechanism used to implement the random allocation sequence (such as sequentially numbered containers), describing any steps taken to conceal the sequence until interventions were assigned | Page 8, Lines 175-177 |
| Implementation | 10 | Who generated the random allocation sequence, who enrolled participants, and who assigned participants to interventions | Page 8, Lines 165-167, Page 8 Lines 172-173, Page 8 Lines 176-178 |
| Blinding | 11a | If done, who was blinded after assignment to interventions (for example, participants, care providers, those assessing outcomes) and how | Page 8, Lines 174-178 |
|  | 11b | If relevant, description of the similarity of interventions | Page 8, Lines 183-184 |
| Statistical methods | 12a | Statistical methods used to compare groups for primary and secondary outcomes | Page 13, Lines 289-296 |
|  | 12b | Methods for additional analyses, such as subgroup analyses and adjusted analyses | Page 13, Lines 297- Page 14, Lines 302 |
| Results | | | |
| Participant flow (a diagram is strongly recommended) | 13a | For each group, the numbers of participants who were randomly assigned, received intended treatment, and were analysed for the primary outcome | Page 15, Lines 329-331, Fig. 1 |
|  | 13b | For each group, losses and exclusions after randomisation, together with reasons | Page 15, Lines 331-333, Fig. 1 |
| Recruitment | 14a | Dates defining the periods of recruitment and follow-up | Page 7, Line 159 |
|  | 14b | Why the trial ended or was stopped | Page 7, Lines 159-160 |
| Baseline data | 15 | A table showing baseline demographic and clinical characteristics for each group | Pages 16-17, Table 1 |
| Numbers analysed | 16 | For each group, number of participants (denominator) included in each analysis and whether the analysis was by original assigned groups | Pages 16-17, (Table 1), Page 17 (Table 2) & S2 file, Page 19 (Table 3), Page 21 (Table 4) & S4 file |
| Outcomes and estimation | 17a | For each primary and secondary outcome, results for each group, and the estimated effect size and its precision (such as 95% confidence interval) | Page 17 (Table 2) & S2 file, Page 19 (Table 3), Page 21 (Table 4) & S4 file |
|  | 17b | For binary outcomes, presentation of both absolute and relative effect sizes is recommended | Page 17 (Table 2) & S2 file, Page 19 (Table 3), Page 21 (Table 4) & S4 file |
| Ancillary analyses | 18 | Results of any other analyses performed, including subgroup analyses and adjusted analyses, distinguishing pre-specified from exploratory | N/A |
| Harms | 19 | All important harms or unintended effects in each group (for specific guidance see CONSORT for harms) | Page 20, Lines 382-Page 21, Lines 403, Page 21 (Table 4) & S4 file |
| Discussion | | | |
| Limitations | 20 | Trial limitations, addressing sources of potential bias, imprecision, and, if relevant, multiplicity of analyses | Page 24, Lines 458-463 |
| Generalisability | 21 | Generalisability (external validity, applicability) of the trial findings | Page 24, Lines 462-468 |
| Interpretation | 22 | Interpretation consistent with results, balancing benefits and harms, and considering other relevant evidence | Page 22- Page 24, Lines 468 |
| Other information | | |  |
| Registration | 23 | Registration number and name of trial registry | Page 3, Line 49 |
| Protocol | 24 | Where the full trial protocol can be accessed, if available | N/A |
| Funding | 25 | Sources of funding and other support (such as supply of drugs), role of funders | Page 9, Lines 185-187 & Page 24, Lines 470-479 |

Citation: Schulz KF, Altman DG, Moher D, for the CONSORT Group. CONSORT 2010 Statement: updated guidelines for reporting parallel group randomised trials. BMC Medicine. 2010;8:18.
© 2010 Schulz et al. This is an Open Access article distributed under the terms of the Creative Commons Attribution License (<http://creativecommons.org/licenses/by/2.0>), which permits unrestricted use, distribution, and reproduction in any medium, provided the original work is properly cited.

*We strongly recommend reading this statement in conjunction with the CONSORT 2010 Explanation and Elaboration for important clarifications on all the items. If relevant, we also recommend reading CONSORT extensions for cluster randomised trials, non-inferiority and equivalence trials, non-pharmacological treatments, herbal interventions, and pragmatic trials. Additional extensions are forthcoming: for those and for up-to-date references relevant to this checklist, see [www.consort-statement.org](http://www.consort-statement.org/).
