# Supplementary material for: Low-dose aspirin is not effective as an adjunct treatment for HIV infection among people living with HIV on dolutegravir-based antiretroviral therapy: A randomised double-blind, parallel-group placebo-controlled trial
Source: PLoS One. 2025 Aug 29;20(8):e0331087. doi: 10.1371/journal.pone.0331087 (PMC12396663; doi:10.1371/journal.pone.0331087)
Supplement: S3 File — (PDF) [file pone.0331087.s014.pdf]

MUHIMBILI UNIVERSITY OF HEALTH AND ALLIED SCIENCES

SCHOOL OF MEDICINE

DEPARTMENT OF CLINICAL PHARMACOLOGY

THE EFFECT OF ASPIRIN ON HUMAN IMMUNODEFICIENCY VIRUS (HIV) DISEASE PROGRESSION AMONG HIV- INFECTED INDIVIDUALS INITIATING ANTI- RETROVIRAL THERAPY.

PhD proposal

by

TOSI MICHAEL MWAKYANDILE

(MD, MSc. CLINICAL PHARMACOLOGY)

Supervisors:

Prof. Eligius F. Lyamuya- Department of Microbiology and Immunology, School of Medicine, MUHAS.

Dr. Grace A. Shayo- Department of Internal Medicine, School of Medicine, MUHAS.

Contents

Definitions.......viii
ABSTRACT....... ix
1. INTRODUCTION....... 1
1.1 Background....... 1
1.2 Problem statement....... 7
1.3 Conceptual framework....... 8
1.4 Rationale....... 9
1.5 Research questions....... 10
1.6 Study objectives....... 11
1.6.1 Broad objective....... 11
1.6.2 Primary objective....... 11
1.6.3 Secondary objectives....... 11
1.7 Literature review....... 12
2. METHODOLOGY....... 16
2.1 Study design....... 16
2.2 Study design diagram....... 16
2.3 Study population....... 16
2.4 Number of Subjects....... 16
2.5 Expected duration of study population....... 16
2.6 Study Area....... 17
2.7 Primary and Secondary Outcome Measures....... 17
2.8 Study treatments....... 18
2.8.1 Treatment Groups....... 18
2.8.1.1 Description of study drugs....... 18
2.8.1.2 Dosage and Route of Administration....... 18
2.8.1.3 Dose modification....... 18
2.8.2 Dispensing and Study Drug Accountability....... 18
2.8.3 Measurement of subject compliance....... 19
2.8.4 Excluded medications and treatments....... 19
2.9 Subject enrolment and randomization....... 20
2.9.1 Recruitment....... 20
2.9.2 Eligibility Criteria....... 21

2.9.2.1 Inclusion Criteria 21
2.9.2.2 Exclusion Criteria 21
2.9.3 Randomization Procedures 21
2.9.4 Blinding Arrangements 22
2.9.5.1 On Study 22
2.9.5.2 Following Completion of the Study 22
2.9.6.1 Reasons for withdrawal 22
2.9.6.2 Handling of withdrawals and losses to follow- up 23
2.9.6.3 Replacements 23
2.10 Study Visits and Procedures schedule 23
2.11 Clinical and laboratory assessments 27
2.12 Adverse event reporting 30
2.12.4.1 SAEs 32
2.12.4.2 SUSARs 32
2.13 Statistical Methods 33
2.14 Data Management 34
2.15 Ethical considerations and approval 34
2.16 Study limitations and mitigation measures 36
2.17 Dissemination plan 37
3. BUDGET AND ITS JUSTIFICATION 38
3.1 Budget 38
3.2 Budget justification 39
4. WORK PLAN 41
5. REFERENCES 43
6. APPENDICES 49
6.1 Appendix 1- Informed Consent Forms 49
6.1.1 Informed consent form 49
6.1.2 Fomu ya Ridhaa 55
6.2 Appendix 2 – Case Report Form for Visit 0 61
6.3 Appendix 3 – Case Report Form for Subsequent visits 69
6.4 Appendix 4 – Death Report Form 76
6.5 Appendix 5 – Prohibited medications during the study 77
6.6 Appendix 6 – Definitions of some terms 79

6.7 Appendix 7 – Formula for calculating eGFR 80
6.8 Appendix 8 – Bleeding disorders 81
6.9 Appendix 9 – Casuality and assessment of severity- Adverse Events 82
6.10 . Appendix 10 – Procedure for PBMC separation 84

List of Abbreviations

AE- Adverse Events

AIDS- Acquired Immunodeficiency Syndrome

AMI- Acute Myocardial Infarction

ART- Anti- retroviral treatment/ therapy

ARV- Anti- retroviral

ASA- Acetyl Salicylic Acid

BMI- Body Mass Index

COX- Cyclo- oxygenase

CPL- Central Pathology Laboratory

CRFs- Case Report Forms

CTCs- Care and Treatment Centres

DSMB- Data and Safety Monitoring Board

FBP- Full Blood Picture

GCLP- Good Clinical and Laboratory Practice

GCP- Good Clinical Practice

HAART- Highly Active Antiretroviral Therapy

hCG- human Chorionic Gonadotrophin

HIV- Human Immunodeficiency Virus

ICH- International Council for Harmonization of Technical Requirements for Pharmaceuticals for Human Use

JSI- John Snow, Inc.

LFT- Liver Function Test

MCRL- MUHAS Clinical Research laboratory

MDH- Management and Development for Health

MNH- Muhimbili National Hospital

MoHCDGEC- Ministry of Health, Community Development, Gender, Elderly and Children

MPAs- Monocyte- Platelet Aggregates

MUHAS- Muhimbili University of Health and Allied Sciences

NIMR- National Institute for Medical Research

NSAIDs- Non-Steroidal Anti- Inflammatory Drugs

PAI- PharmAcess International

PASADA- Pastoral Activities and Services for People with AIDS Dar es Salaam Archdiocese

PBMCs- Peripheral Blood Mononuclear Cells

PI- Principal Investigator

PLHA- People Living with HIV and AIDS

PSGL- 1- P- Selectin Glycoprotein Ligand 1

RFT- Renal Function Test

SAEs- Serious Adverse Events

sCD14- Soluble CD14

SOPs- Standard Operating Procedures

sP- selectin- Soluble P- selectin

SUSAR- Suspected Unexpected Serious Adverse Reaction

TFDA- Tanzania Food and Drug Authority

TNF- α- Tumour Necrosis Factor- alpha

UNAIDS- Joint United Nations Programme on HIV/ AIDS

WHO- World Health Organization

Definitions.

1. **Adherence to ART** will be defined as greater than 90% adherence by pill count.
2. **Adverse Event (AE)** will be defined as any untoward medical occurrence in a patient enrolled into this study regardless of its causal relationship to study treatment.
3. **Serious Adverse Event (SAE)** will be defined as any AE that results in death; or is immediately life threatening; or requires inpatient hospitalization; or requires prolongation of existing hospitalization; or results in persistent or significant disability/incapacity; or is a congenital anomaly/birth defect.
4. **Non-Serious Adverse Event** will be defined as any AE that does not result in death; or is not immediately life threatening; or does not require inpatient hospitalization; or does not require prolongation of existing hospitalization; or does not result in persistent or significant disability/incapacity; or is not a congenital anomaly/birth defect.
5. **Suspected Unexpected Serious Adverse Reaction (SUSAR)** will be defined as any SAE that is both suspected to be related to the study treatment and is unexpected (i.e. not consistent with applicable product information).
6. **Morbidity** will be defined as number of health facility/ hospital visits for medical attention/ care because of illness and/or number of health facility/ hospital admissions.
7. **All- cause mortality** will be defined as confirmed death of any cause with certification of death by medical practitioner or a verbal or telephone confirmation of death from a relative or friend.
8. **Clinical conditions associated with death** will be defined as those clinical conditions diagnosed within one month prior to or at time of death or were thought to be related to death after review of the medical records.
9. **Low- dose- aspirin (ASA)** will be defined as ASA dosages between 75mg and 325mg taken every day.
10. **Compliance to ASA** will be defined as adherence to ASA therapy for at least 22 days in a month.

ABSTRACT

Background; Despite the introduction of antiretroviral (ARV) therapy, HIV is still a public health problem in Eastern and Southern Africa. The wide spread use of the ARV drugs has improved the life expectancy of people living with HIV and/ or acquired immunodeficiency syndrome (AIDS). As a result, there has been an increase in the prevalence of and mortality from non- AIDS complications such as non- AIDS defining cancers, liver, pulmonary and cardiovascular diseases. The increase in the non – AIDS complications especially the cardiovascular disease risk is linked to platelet and immune activation. Antiretroviral therapy (ART) does not completely abolish the immune activation and the platelet activation making it a necessity to find additional therapy to the conventional ART therapy that will decrease the occurrence of these non- AIDS complications and their associated morbidities. Aspirin or acetyl salicylic acid (ASA) has shown promise as such an additional drug. In addition, ASA appears to have an array of beneficial effects in the HIV infected individuals. ASA is reported to cause a reduction and maintenance in HIV load, significantly increase CD4 counts and halt the clinical HIV disease progression. Literature shows that additional therapy to ARV drugs may compromise adherence to ART among HIV- infected individuals. However, when addition of a pill is associated with significant benefit, the addition of a pill or pills may be justifiable. For example, addition of medication for opportunistic infection, methadone maintenance therapy and antidepressants has been reported to improve adherence to ART among HIV- infected patients in some studies. Therefore, considering the reported benefits of ASA in the HIV- infected population, it is important to study the effect of the addition of ASA on both the HIV disease progression and the adherence to ARV drugs.

Objective(s): To determine the effect of low dose of ASA on HIV disease progression and adherence to anti- retroviral therapy among HIV-infected individuals initiating ARV therapy.

Materials and Methods: The study will be a phase IIA randomized double blind, placebo-controlled trial with two study groups involving 454 antiretroviral drugs naïve adult patients initiating ART who are attending HIV clinics at **Mwananyamala** and Mbagala Rangi Tatu in Dar es Salaam. Patients in the two study groups will be receiving, in addition to ARV drugs, 75 mg ASA or placebo once daily for six months. They will be followed up monthly for a period of six months. Blood samples will be collected at baseline for all the tests and at months 6 for lipid profile, full blood picture (FBP), renal function test (RFT) and liver function test (LFT), at

months 3 and 6 for CD4 count, at months 2, 3 and 6 for HIV viral load and at months 3 and 6 for plasma levels of soluble CD14 (sCD14), soluble P- selectin (sP- selectin) and for markers of T cell activation and exhaustion. Urine will be collected to exclude pregnant women. The primary outcome will be virological response i.e. the proportion of patients in the two arms reaching viral loads of < 50 copies/ millilitre at months 2, 3 and 6 (indicating viral suppression) and > 1000 copies/ millilitre at month 6 (indicating virologic failure). The secondary outcomes will be immunologic response measured by CD4 count and clinical responses measured by morbidity and death from any cause. Other secondary outcome measures will be plasma levels of sCD14 and sP- selectin, percentage of activated T cells (CD38 positive and HLA-DR positive T cells), percentage of exhausted T-cells (PD-1 positive T cells), percentage adherence to ART and compliance to study medications, adverse events. Data will be analysed by intention to treat. The study outcomes in the two treatment groups will be compared using Chi- square, Fisher's exact or student's t tests and descriptive statistics will be used where appropriate.

Budget of the study; The estimated budget for the proposed study 123,722,026.40 Tshs

1. INTRODUCTION

1.1 Background

HIV infection continues to be a global public health problem. According to the Joint United Nations Programme on HIV/ AIDS (UNAIDS) data of 2017, approximately 36.7 million people were living with HIV worldwide with the Eastern and Southern Africa being a highly-burdened region contributing more than half of the cases in 2016. Additionally, the Eastern and Southern Africa had the highest percentage of the new HIV infections compared to the other regions in the world(1) leading to more burden of HIV infection in the future. Although there has been a global decline in AIDS- related deaths from 2004 to recent years and that the Eastern and Southern Africa had the sharpest decline, deaths from HIV/ AIDS are still high in this part of the world(1).

In Tanzania, by 2016, a total of 1.4 million people were living with HIV and AIDs- related deaths were 31000 which is a 50% decline from about five years previously. However, this number is still high(1). The decrease in AIDS- related mortality, in Tanzania and elsewhere, is mostly a result of the introduction and widespread use of the highly active antiretroviral therapy (HAART)(2).

Since the introduction of ART and the highly active antiretroviral therapy, people living with HIV and AIDS (PLHA) are now living longer than before (have improved life expectancy)(2,3). The improved life expectancy is a result of stable or improved CD4 counts, suppressed HIV loads and clinical benefits provided by the ART(2). Despite the improved life expectancy, when compared to the general population, PLHA still die earlier(3). This may be because, PLHA are living longer to experience age-related co- morbidities and that there has been an increase in the prevalence of and mortality from non-HIV/AIDS complications such as non- AIDS defining malignancies, hepatic, pulmonary and cardiovascular diseases among them(2).

Cardiovascular disease has become of great importance as a cause of morbidity and mortality among the HIV- infected patients(4–6). Particularly, cardiovascular disease is now one of the top causes of deaths in the HIV- infected population (7,8). The risk of cardiovascular diseases such as myocardial infarction (MI) and sudden cardiac death in both the treated and untreated HIV-infected individuals is significantly higher than in the HIV- non- infected ones. Among the HIV-infected population, the risk is higher for those on antiretroviral treatment compared to those who are treatment naïve(9) suggesting that the metabolic adverse effects of the ARV drugs may have a role. Indeed, the incidence of cardiovascular disease is reported to be higher in the ART-

successfully treated HIV- infected patients than the HIV non- infected ones(10). However, the mechanism of the increased cardiovascular disease risk among HIV- infected is still not completely understood. Studies have reported an interplay of many other factors apart from the metabolic adverse effects of the ARV drugs. Some of such factors are the high prevalence of traditional risk factors for cardiovascular disease (smoking, hypertension, diabetes and dyslipidaemia)(11) within the HIV- infected individuals, the replication of HIV itself and a low CD4 count (12,13). Prolongation of life is also a postulated factor as people live longer enough to develop cardiovascular diseases. Additionally, chronic immune activation is partially attributed to the increased risk for cardiovascular disease in PLHA. Immune activation (of both the innate and adaptive immune response) is reportedly higher in the HIV infected population than the HIV- non infected population and is said to result from low level viral replication, adipose tissue dysfunction, coinfection and translocation of microbial products from a damaged gut.

Among activation of other cells of the immune system, less clearly, T cell activation has been reported to play a role in the development of cardiovascular disease in the HIV- infected individuals. The levels of T cell activation and T cell exhaustion are defined by the expression of cellular markers CD38, HLA- DR and PD-1, respectively. HIV- infected individuals exhibit increased markers of T cell activation and studies have shown that the ARV naïve HIV- infected individuals have higher frequencies of activated T cells than those on ART with viral suppression. Furthermore, it is reported that even with successful treatment with ART, percentages of activated T cells remain higher than in the HIV uninfected population(14–16). Therefore, T cell activation may be one of the factors that can be linked to increase risk for cardiovascular disease in HIV- infected individuals. Indeed, it has been shown that T cell activation is associated with subclinical atherosclerosis among PLHA(17).

Apart from T cell activation, monocyte activation also is associated with vascular disease in the HIV- infected patients(18–20). Monocytes are persistently activated in HIV- infected patients. Activated monocytes are associated with HIV pathogenesis and progression and development of cardiovascular disease. Following activation, monocytes produce, among other molecules, sCD14 by enzymatic shedding of CD14 from the plasma membrane. The liver also secretes sCD14 as an acute- phase protein in response to interleukin- 6. In the HIV- non- infected elderly individuals and those with chronic kidney disease, sCD14 has been linked to cardiovascular diseases such as

MI(21,22). Likewise, in the HIV- infected sCD14 is reportedly associated with cardiovascular disease(23). Plasma levels of sCD14 are higher in the HIV- infected patients as compared to those in the general population(16,24). Different studies have reported that sCD14 levels do not return to normal values even in the ART- virologically suppressed patients(24–27). Elevated levels of sCD14 in the PLHA may reflect the extent of monocyte activation (as part of the chronic immune activation) among these individuals. All these data therefore, suggest that monocyte activation (as a component of immune activation) together with the associated biomarker (sCD14) may play a role in the pathogenesis of HIV- related cardiovascular disease.

Different causes for monocyte activation in the HIV- infection have been put forward and activated platelets is one of them. There is increased platelet activation in the HIV- infected individuals(28) and activated platelets express, among other molecules, P- selectin to which monocytes bind via P-selectin glycoprotein ligand-1 (PSGL-1) to form monocyte- platelet aggregates (MPAs). P-selectin is one of the several adhesion molecules expressed by platelets whose major function is to facilitate the process of clot or thrombi formation. In the platelets, P- selectin is stored in the alpha granules and when platelets are activated, P- selectin quickly moves to the cell surface of the platelets (P- selectin expression). The expression of P- selectin is short lived and the molecule is degraded or recycled following its quick internalization in the cell. P- selectin can be secreted into the circulation in a soluble form giving the sP- selectin. It is reported that elevated levels of sP-selectin are associated with atherosclerosis and thrombosis and are predictive of future adverse cardiovascular events such as MI and stroke(29–31).

It has been shown that in the HIV- infected individuals' plasma levels of sP- selectin are higher than in the HIV non- infected population (27) (secondary to the increased platelet activation)and that the cardiovascular disease risk is increased compared to the non-HIV- infected. There is inconsistency in data with regards to the effect of ART to platelet activation (the levels of sP-selectin). In some reports, sP- selectin levels normalize to non- HIV-infected levels with ART(27) while in some reports the levels do not decrease(32).

Therefore, residual platelet activation and immune activation (as reflected by persistent levels or expression of the respective biomarkers with ART) may be a major shortcoming of the ART, as platelet activation and immune activation are implicated in the increased cardiovascular in PLHA. Decreased platelet and immune activation together with their associated biomarkers may mean

decreased cardiovascular disease risk, reduced HIV disease progression, decreased morbidity and mortality among the HIV- infected population. The use of ARV drugs alone too, results into reduced HIV disease progression, decreased morbidity and mortality through increasing CD4 counts, decreasing both HIV loads and the incidence of opportunistic infections. However, the ARV drugs only partially reduce platelet and immune activation leaving elements of platelet and immune activation which may be associated with cardiovascular disease risk in PLHA**.** Due to this possible drawback of ARV drugs on immune activation and platelet activation, an additional drug is necessary for effective management of the non- infectious co morbidities in HIV infection.

Aspirin or acetyl salicylic acid (ASA) is a drug with antiplatelet and anti-inflammatory properties. The acute role of ASA on the increased platelet and immune activation in the HIV- infected population has been explored with conflicting findings. In one study, low- dose ASA appeared to lessen both platelet and immune activation and the associated biomarkers in the HIV- infected population in one study(16) while in another study it did not affect biomarkers of immune activation(33). Data on the long-term use of ASA and its effect on platelet and immune activation and consequently, on morbidity and mortality among HIV-infected population are deficient.

ASA was first synthesized in 1897 by chance as a chemist was trying to find an alternative remedy for his father’s rheumatism. ASA is one of the long-time known drugs that has been widely used and studied. It belongs to the class of the non- steroidal anti- inflammatory drugs (NSAIDs) and it has a wide range of therapeutic effects depending on the dose given(34).

At low doses, ASA brings about its antiplatelet effects making it a suitable drug for primary and secondary prevention of cardiovascular disease. For anti-inflammatory and analgesic effects, higher doses of ASA are required(34). All these effects of ASA are said to be mediated through inhibition of both isoforms of the cyclo-oxygenase (COX) enzyme affecting the synthesis of prostaglandins, prostacyclins and thromboxanes.

Interestingly, by not so well-established mechanisms ASA appears to have an array of beneficial effects in the HIV infected individuals. ASA is reported to halt the clinical HIV disease progression (35). ASA has been shown to significantly increase CD4 counts, an effect appreciable in a few months to a year of ASA use at moderate to high doses in the HAART naïve patients(36). ASA has also been observed to cause a reduction and a maintenance in HIV load(35,37).

The use of ASA in the HIV- infected individuals is associated with other effects like decrease in p24 antigen, decrease in tumor necrosis factor alpha (TNF-α), improvement in body weight (together with Body Mass Index [BMI]) and hemoglobin levels(35,36). These effects of ASA in one way or another may contribute to improvement in quality of life, meaning decreased morbidity and/or mortality associated with HIV/ AIDS.

Despite these useful effects, there are risks associated with the use of ASA such as gastrointestinal bleeding and rarely cerebral bleeding which are reported non- fatal or life-unthreatening. It is reported however that these adverse effects are more likely associated with high doses than with low doses and that duration of ASA use does not appear to increase the risk for the adverse effects(38). Thus, the use of ASA at low dose may still be advantageous in the HIV- infected population considering the above-mentioned beneficial effects. Therefore, there is still a need to explore the long- term effects of ASA on morbidity, mortality and the HIV disease progression in general among PLHA.

As it goes with any medications, to achieve successful treatment outcomes medication adherence is very essential. In the management of HIV and AIDS, adherence to ART is crucial for therapeutic outcome since it is a major predictor of virologic suppression, immunologic recovery, disease progression , resistance to ARV drugs, disease progression and death(39–44).

Adherence to ARV medications can be evaluated by various methods such as self-reports using validated questionnaires, patient diaries, pharmacy records, plasma levels, pill counts, electronic monitors and others. For adherence to ART, there is no gold standard method as each method has its merits and shortcomings. Measuring adherence to ART by pill count has been employed in many investigations. Although adherence by pill counts overestimates adherence, it correlates with adherence by electronic monitors ,which is an objective way to measure adherence, and viral load measurements(44–46). In one study, pill count adherence was not significantly different from adherence by self- reports, plasma levels and electronic monitors(44).

Adherence to ARV drugs may be affected by a number of factors including adverse drug effects/ reactions due to drug- drug interactions and high pill burden. There are no reported clinically significant drug-drug interactions between ASA and any of the drugs in ART(47) and hence there are no anticipated adverse drug reactions to compromise adherence when ASA is given together with the ARV drugs. High pill burden of the ARV regimen or from co- medication is one of the

many factors that are associated with non- adherence or low adherence(48,49). Adding ASA, which is not co- formulated with the ARV drugs, to the conventional ARV therapy and other prophylactic drugs for opportunistic infections which are normally given in the management of HIV/ AIDS may increase pill burden among the HIV-infected individuals, consequently affecting their adherence to ARV therapy, which is the backbone of their therapeutic management. Therefore, the proposed study will look at the level of adherence to ARV drugs among HIV-infected individuals taking their ARV drugs and their routine prophylactic drugs against opportunistic infections and the added ASA or placebo.

1.2 **Problem statement**

Despite the introduction of ARV therapy, HIV is still a public health problem in Eastern and Southern Africa(1). The wide spread use of the ARV drugs has improved the life expectancy of people living with HIV and/ or AIDS. As a result, there has been an increase in the prevalence of non- AIDS complications such as non- AIDS defining cancers, liver, pulmonary and cardiovascular diseases(2).

The increase in the non – AIDS complications especially the cardiovascular disease risk is linked to platelet and immune activation(18–20,29–31). The ART does not completely abolish the immune activation and the platelet activation(24–27,32) making it a necessity to find additional therapy to the conventional ART therapy, that will decrease the occurrence of these non- AIDS complications and their associated morbidities. ASA promisingly shows to be that additional drug. Therefore, the beneficial effects of ASA in the HIV- infected individuals need to be investigated.

Literature shows that additional therapy to ARV drugs may compromise adherence to ARV drugs among HIV- infected individuals(48). However, when addition of a pill is associated with significant benefit, the addition of a pill or pills may be justifiable. For example, addition of medication for opportunistic infection, methadone maintenance therapy and antidepressants has been reported to improve adherence to ART among HIV- infected patients in some studies(50–52). Therefore, considering the reported benefits of ASA in the HIV- infected population, it is important to study the effect of the addition of ASA on both the HIV disease progression and the adherence to ARV drugs.

1.3 Conceptual framework

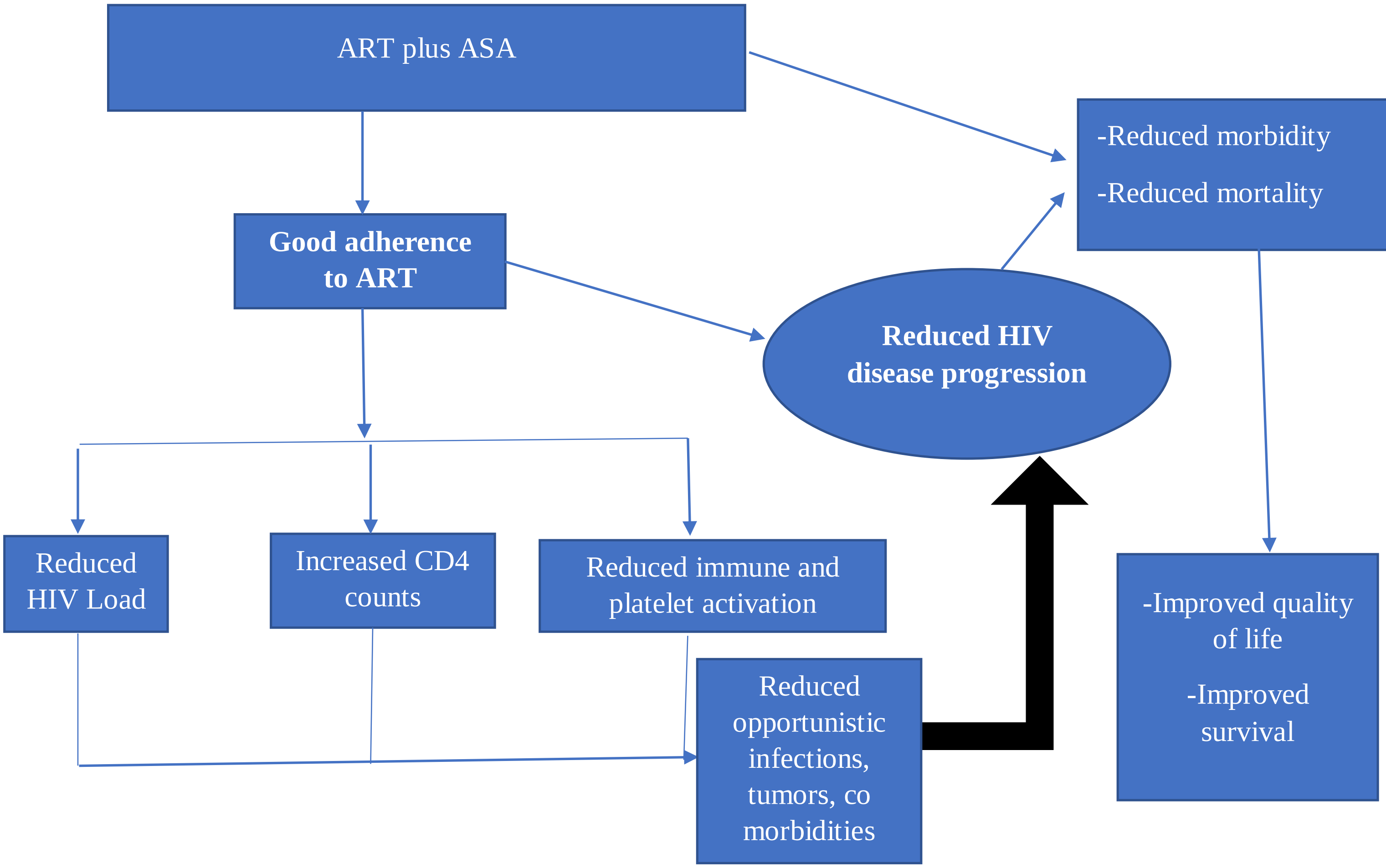

Figure 1.1: Conceptual Framework

HIV disease progression is influenced by a range of factors. ART slows HIV disease progression and alters its course significantly, however, for therapeutic success adherence to ART must be nearly perfect to achieve a sustained virologic suppression. Plasma viral load and CD4 cell count are important laboratory measures of the rate of progression. Short term use of ASA is reported beneficial to PLHA as it increases CD4 cell count, decreases plasma viral load and reduces platelet and immune activation. Introducing ASA to the conventional ARV regimen may maximise these benefits without compromising the adherence to ART, which would then lead to reduced morbidity and mortality and hence improved quality of life and survival among PLHA. The proposed study aims at determining the effects of ASA on HIV disease progression among HIV- infected patients initiating ART.

1.4 Rationale

Since initiation of treatment and care of HIV-infected patients in Tanzania and scaling-up of treatment to reach populations in all settings in the country, parallel efforts have been made to improve compliance to treatment. As observed in the foregone literature review it is prudent to establish the potential benefit of adding ASA in the current ARV treatment regimen in the country and also investigate the impact of adding ASA on treatment adherence. The goal is to establish strategies for enhancing ART impact and further improving treatment outcome. ASA is a cheap drug that is easily available in Tanzania and once its beneficial effect is confirmed its use in ART therapy can be easily implemented in the current National HIV treatment programme.

Findings from this proposed study will provide preliminary data for policy makers especially in the Ministry of Health, Community Development, Gender, Elderly and Children (MoHCDGEC) on the possibility of adding ASA to the conventional ARV treatment in the HIV- infected individuals to reduce morbidity and mortality. Hence, this proposed study will provide preliminary data for future consideration for ASA use in HIV care and treatment.

1.5 Research questions

1. What effect does ASA have on HIV viral load among HIV- infected individuals initiating ARV therapy?
2. What effect does ASA have on CD4 counts among HIV- infected individuals initiating ARV therapy?
3. What effect does ASA have on plasma level of immune activation biomarker (sCD14) among HIV- infected individuals initiating ARV therapy?
4. What effect does ASA have on plasma level of platelet activation biomarker (P-selectin) among HIV- infected individuals initiating ARV therapy?
5. What effect does ASA have on T cell activation biomarkers (CD38 and HLA-DR) among HIV- infected individuals initiating ARV therapy?
6. What effect does ASA have on T cell exhaustion biomarker (PD-1) among HIV-infected individuals initiating ARV therapy?
7. What effect does ASA have on morbidity among HIV- infected individuals initiating ARV therapy?
8. What effect does ASA have on all- cause- mortality among HIV- infected individuals initiating ARV therapy?
9. What is the level of adherence to ARV therapy among HIV- infected individuals initiating ARV therapy and ASA or placebo?

1.6 Study objectives

1.6.1 Broad objective

- To determine the effect of low dose of ASA on HIV disease progression and adherence to anti- retroviral therapy among HIV-infected individuals initiating ARV therapy.

1.6.2 Primary objective

The primary objective of the proposed study is:

- To compare the HIV viral loads measured at 2, 3 and 6 months among HIV- infected individuals after initiating ARV therapy alone or ARV therapy and 75mg ASA.

1.6.3 Secondary objectives

The secondary objectives of the proposed study are:

1. To compare the CD4 counts measured at 3 and 6 months among HIV- infected individuals after initiating ARV therapy alone or ARV therapy and 75mg ASA.
2. To compare the plasma levels of immune activation biomarker (sCD14) measured at 3 and 6 months among HIV- infected individuals after initiating ARV therapy alone or ARV therapy and 75mg ASA.
3. To compare the plasma levels of platelet activation biomarker (P- selectin) measured at 3 and 6 months among HIV- infected individuals after initiating ARV therapy alone or ARV therapy and 75mg ASA.
4. To compare percentage of activated T cells (CD38 positive and HLA-DR positive T cells) measured at 3 and 6 months among HIV- infected individuals after initiating ARV therapy alone or ARV therapy and 75mg ASA.
5. To compare percentages of exhausted T cells (PD1 positive T cells) measured at 3 and 6 months among HIV- infected individuals after initiating ARV therapy alone or ARV therapy and 75mg ASA.
6. To compare morbidity among HIV- infected individuals initiating ARV therapy alone or ARV therapy and 75mg ASA.
7. To compare all-cause-mortality among HIV- infected individuals initiating ARV therapy alone or ARV therapy and 75mg ASA.
8. To determine level of adherence to ARV therapy among HIV- infected individuals initiating ARV therapy and ASA or placebo.

1.7 Literature review

There are a few studies showing the effect of ASA on HIV disease progression. In one 12- month, three- arm, placebo-controlled pilot study conducted in Zimbabwe investigating the use of ASA in the management of HIV disease, it was shown that 300 mg of ASA given four times a day to ARV naïve HIV- infected patients resulted into no increase in HIV viral load during the follow up period. Unlike in the placebo arm where there was an increase in the HIV viral load(37). In another study also conducted in Zimbabwe but among ARV naïve AIDS patients, there was a decrease in HIV viral load in the first 6 months of a combination of 300 mg ASA four times a day together with chloroquine and micronutrients. When chloroquine was stopped and these patients continued with ASA and micronutrients for an additional 3 months, there was a median HIV viral load decline at the end of 9 months relative to baseline. This decline cannot be ascertained to ASA alone. In the same study however, notwithstanding the net decline some patients' HIV viral load increased(35). Findings from these studies are contradictory and that effects on HIV viral load from the latter cannot be completely ascertained to ASA, it is therefore necessary to conduct further studies to show the effect of ASA on HIV load.

Apart from having effects on the HIV viral load, ASA has also immunologic benefits i.e. it has been observed to increase CD4 count in HIV- infected individuals with or without AIDS. The CD 4 T cells are the primary target cells for the HIV. Therefore, CD4 count reflects the extent of immune depletion and hence it is a direct marker for the risk of morbidity and/ or mortality in HIV- infected individuals. Any agent that can increase the CD4 count is likely to reduce the risk of morbidity and/ or mortality in the HIV- infected population. In a study done in Zimbabwe investigating the use of ASA in the management of HIV disease among ARV naïve patients, it was shown that ASA, in divided doses, of 1.2 grams a day resulted in an increase of CD4 count in a period as short as 24 weeks. However, this increase was followed up by an insignificant drop of the CD4 count at 12 months but still higher than the baseline count (37). In another study, again conducted in Zimbabwe, ARV naïve patients with CD4 counts less than 200 cells/milliliter had their CD4 counts increased within 3 months of 600 mg two times daily use of a combination of ASA and micronutrients. In this study however, it was not possible to ascertain the increased CD4 count to ASA or micronutrients alone (35). In another study done in Nigeria, similarly a combination of ASA and other nutrients resulted into a non-significant increase in CD4 counts

(36). In all these studies however, the sample sizes were small. In the proposed study, a relatively larger sample size of HIV- infected patients initiating ARV therapy is going to be involved.

Literature shows that among other factors, platelet and immune activation have a higher contribution to the increased cardiovascular disease risk in the HIV- infected population(18–20,29–31). Additionally, literature also shows that ARV therapy alone can only partially decrease the exaggerated immune activation and its associated biomarkers in this population(24–27). There is inconsistency in data with regards to the effect of ART to platelet activation and its associated biomarkers. In some reports, levels of platelet activation biomarker normalize to non- HIV-infected levels with ART(27) while in some reports the levels do not decrease(32).
In an effort to find an add-on-therapy to the ARV therapy, studies on effects of ASA on platelet and immune activation have been conducted since ASA is both an antiplatelet and anti-inflammatory drug.

Two studies, both done in the United States (US) by O' Brien M *et al* have shown conflicting findings. In the first study, 81 mg of daily ASA for a week among virologically suppressed HIV-infected individuals on ARV therapy resulted into diminished levels of biomarkers of platelet and monocyte activation ((sP- selectin and sCD14 respectively) and diminished expression of biomarkers of T cell activation(CD38 and HLA-DR) reflecting a decrease in platelet and immune activation (16). In the other study, however, ASA at doses of 300 mg or 100 mg daily for 12 weeks among ARV therapy- suppressed HIV- infected individuals did not appear to have any effect on the levels of biomarkers of monocyte activation (sCD14) and T cell activation and exhaustion(CD38, HLA-DR, PD-1)(33). Due to this controversy, the proposed study secondarily aims at studying the long- term effects of ASA on platelet activation, monocyte activation and T cell activation and exhaustion and their respective biomarkers (sP- selectin, sCD14, CD38, HLA-DR, PD-1) among HIV- infected individuals initiating ARV therapy in our settings.

In the general population, ASA has been put in chemo preventive uses especially in oncology, cardiovascular medicine and obstetrics to lower the incidences of a number of diseases. Studies have shown that daily ASA reduce the incidence of colorectal cancer and several other cancers and also reduce metastasis therefore lowering the burden of diseases(53). ASA is also used for the primary and secondary prevention of cardiovascular disease. These benefits have been observed in studies involving the general population therefore, may or may not be seen in the HIV

infected individuals and are appreciable after a long-term use of daily ASA. In the HIV- infected population, although data are limited, short term daily use of ASA has been shown to reduce morbidity.

In one study that was conducted among HIV positive ARV naïve men and women with AIDS, who were given ASA (300mg four times daily), micronutrients and chloroquine for six months then switched to ASA (600mg twice a day) and micronutrients for three months, no opportunistic infections were diagnosed during the last three months of the study. Again, in this study there was no evidence of clinical disease progression in any of the patients. On self-report, all but one patient reported improvements in their conditions(35). In another study involving HAART naïve people living with HIV and AIDS, six months of daily ASA use in combination with selenium and multivitamins resulted in improved quality of life which may be due to reduced morbidity(36). In both these studies, a few numbers of patients were involved and also the effects observed cannot be fully ascertained to ASA as it was given together with other drugs or nutrients. The proposed study therefore, will study the effects of ASA singly on morbidity by looking at number of hospital visits for medical attention/ care because of illness and/or number of hospital admissions among HIV infected patients initiating ARV, for six months.

Although data are scarce, daily use of ASA in the HIV- infected population has shown reduction in morbidity(35). Reduced morbidity may indirectly mean reduced mortality. To the best of my knowledge, there are no studies reporting on the effects of ASA on mortality specifically among the HIV- infected population. Many trials conducted looking at the role of ASA in reducing mortality are focused on cardiovascular diseases and involved the general population(54). The proposed study also aims at looking at the effects of low dose ASA on all-cause-mortality in the HIV-infected population.

It is reported that complexity of the ARV regimen has decreased because of the introduction of fixed dose combination ARV drugs therefore improving adherence to the ARV regimens. However, the HIV- infected patients have other prescription drugs, for other comorbid conditions or prophylaxis for opportunistic infections, something which may limit their adherence to ARV drugs. As far as literature could be searched, there are a few studies which have investigated the influence of co medications on adherence to ARV drugs(48,50–52).

In one prospective observational study that involved HIV- patients determining the influence of co medications on ARV therapy adherence, results showed that the non- adherent patients are the ones who had high number of co medications. This may imply that co medication among HIV- patients may compromise their adherence to ARV drugs(48). In other studies however, introduction of co medications such as antidepressants, medications for opportunistic infections and methadone maintenance therapy for intravenous drug users HIV- infected patients resulted into improvement in adherence to ARV drugs(50–52). In the proposed study the level of adherence to ARV therapy among HIV-infected individuals initiating ARV drugs and also taking ASA or placebo is going to be determined.

2. METHODOLOGY

2.1 Study design

The proposed study will be a phase IIA, double blind, randomized placebo- controlled two sites out- patient based trial with two treatment groups.

2.2 Study design diagram

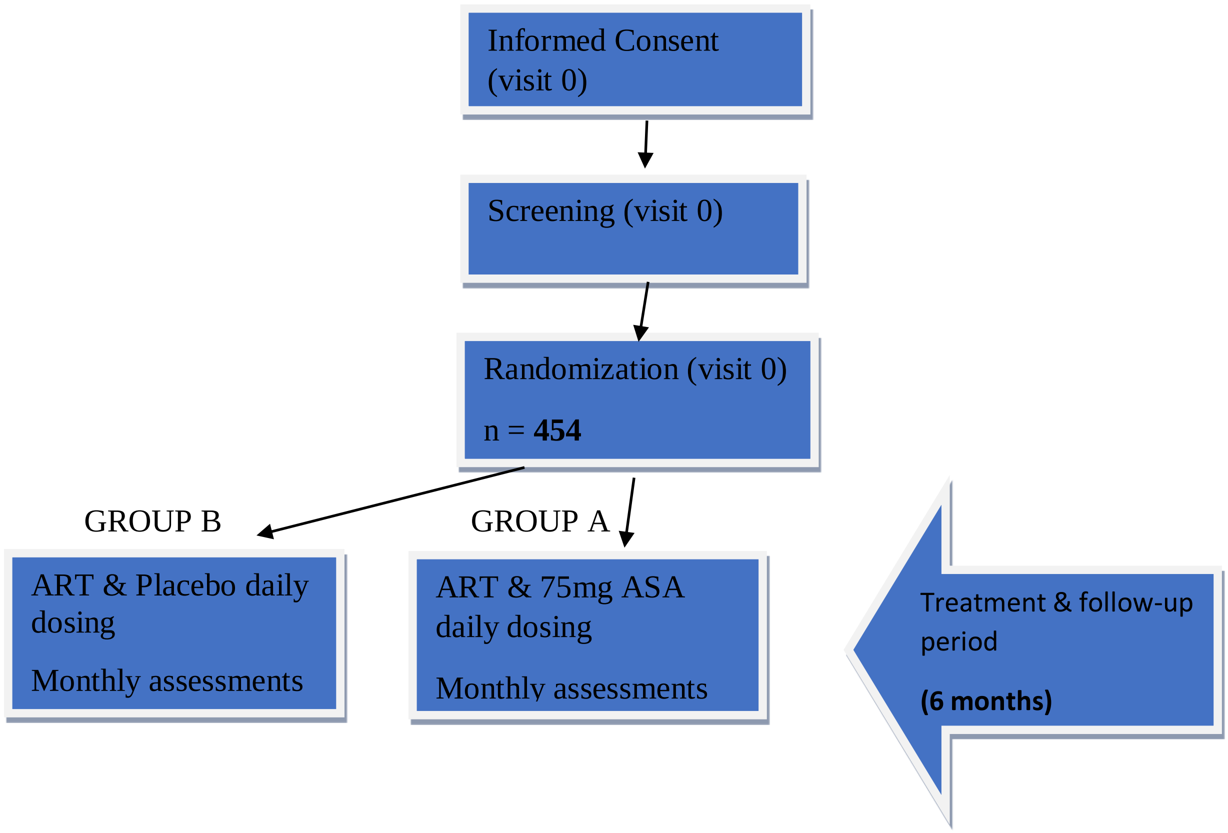

Figure 2.2: Study design flow chart.

2.3 Study population

Newly recruited HIV infected patients initiating ART attending CTCs in Dar es Salaam.

2.4 Number of Subjects.

The study will involve 454 newly recruited HIV infected patients initiating ART attending CTCs in Dar es Salaam who will be equally obtained from each of the two study sites.

2.5 Expected duration of study population

The proposed study is expected to run for 1 year, from start of subject screening to last subject finishing the study. The recruitment period will be six months and each individual

subject will be on study treatment for six months and will also be followed- up for six months.

2.6 Study Area

The proposed study will be conducted in Dar es Salaam which is the largest city in the United Republic of Tanzania. According to the 2012 official census, Dar es Salaam had a population of 4,364,541. Between October 2016 and August 2017, the prevalence of HIV-infection in the region was 4.7% (55). Administratively, Dar es Salaam is divided into five districts namely Ilala, Kinondoni, Temeke, Kigamboni and Ubungo. Within the five districts there are a total of 269 HIV Care and Treatment Centres (CTCs), 235 of them are supported by Management and Development for Health (MDH), and the rest are supported by programs or organizations like Pastoral Activities and Services for People with AIDS Dar es Salaam Archdiocese (PASADA), PharmAcess International (PAI) and John Snow, Inc. (JSI). The cumulative number of persons ever enrolled in care by the end of September 2017 was 317923 persons. On a daily basis, on average 3-5 patients, 1-2 patients and 1 or no patient are newly diagnosed with HIV infection at big, medium and small CTCs, respectively. These clinics operate on six days of a week where Fridays and Saturdays are for paediatric patients and adolescents, respectively, and patients are seen monthly. We propose to conduct the study at the public HIV clinics supported by MDH in Dar es Salaam which include Mbagala Rangi Tatu and **Mwananyamala**. This selection of the study sites is based on the high enrolment of patients and although these clinics are situated in different facilities, they are run similarly.

2.7 Primary and Secondary Outcome Measures

The primary outcome of interest will be virological response. The main virological outcome that will be observed will be the proportion of patients in different arms reaching viral loads of < 50 copies/ millilitre at months 2, 3 and 6 (indicating viral suppression) and > 1000 copies/ millilitre at month 6 (indicating virologic failure).

The secondary outcomes will be immunologic response measured by CD4 count and clinical responses measured by morbidity and death from any cause. With the use of ART alone there is an increment of 30% at 3 months in CD4 count from baseline value(56). With the addition of ASA to the ART, it is expected that the CD4 count will rise by > 30% from baseline value at months 3. Other secondary outcome measures will be plasma levels of sCD14 and sP- selectin, percentage

of activated T cells (CD38 positive and HLA-DR positive T cells), percentage of exhausted T-cells (PD-1 positive T cells), percentage adherence to ART and compliance to study medications, adverse events. The levels of sCD14 and sP- selection are expected to drop to those of non- HIV infected population by 6 months(16).

2.8 Study treatments

2.8.1 Treatment Groups

There will be two treatment groups namely GROUPS A and B. One treatment group will be of participants receiving ART and 75mg of ASA (GROUP A) and the other group will be of participants receiving ART and placebo (GROUP B).

2.8.1.1 Description of study drugs

All the study drugs (ASA and placebo) will be manufactured by *Cosmos Limited* in tablet form and will be packaged in blister packs in boxes. The placebo will have a similar color, shape, taste and size to the active drug. The boxes containing the study drugs will have the following information; batch number, expiry date and the statement "keep all medicines out of the reach of children". Also, arbitrary study letter and month number will be hand written on the boxes.

2.8.1.2 Dosage and Route of Administration

Participants will self-administer one tablet of either 75mg ASA or placebo in addition to their ARV drugs. This will be taken daily at evening times together with their ARV drugs and swallowed whole with plenty of liquid (e.g. a glass of water) preferably after a meal. This should be continued for the six months of treatment and follow- up.

2.8.1.3 Dose modification

There will be no any allowable dose modifications.

2.8.2 Dispensing and Study Drug Accountability

The study drugs will be stored in a pharmacy for each study site in an identified lockable wooden cabinet where temperature will be controlled between 25º Celsius and 30º Celsius. A temperature log will be maintained. Study drug dispensing will be done by a respective pharmacist (CTC staff employed in the study) who will be provided with the randomization schedule. The pharmacist at each site will maintain accurate records of the receipt of all study medication, including dates of

receipt. In addition, accurate records will be kept regarding when and how much study medication is dispensed and used by each patient in the study. At the end of the study, there will be final reconciliation of study drug received, dispensed and consumed. Any discrepancies will be investigated, resolved and documented by the study team. Unused study drug will be destroyed at the end of the study.

2.8.3 Measurement of subject compliance

Unlike with ARV drugs where there are cut- off points to define good or low adherence depending on method used to measure adherence, with ASA there are no. Hence in the proposed study, compliance to study drugs will be defined as adherence to ASA therapy for at least 22 days in a month and will be assessed monthly. This is adopted from a study that was looking at frequency and predictors of non-compliance to ASA therapy in post MI patients where noncompliance was defined as non-adherence to ASA therapy for more than 2 days per week(57). When translated to a month (30 days), then adherence to ASA therapy for at least 22 days in 30 days is compliance.
Assessment of compliance to study drugs will be by pill count. At each visit, patients will be given their study drug refill and will be asked to return with the study drug boxes in the subsequent visit. Patients will be provided with study drug pills to suffice 30 days to take home. To measure compliance, extra tablets to the 30 tablets will be provided at each monthly visit. The number of the extra tablets will be changing so that the number of tablets remaining in each month will not be fixed. This will keep the patients from predicting the number of tablets to remain in the study drug box. Patients' remaining monthly tablets of their study drugs will be counted, by the study doctor, to ascertain compliance at each monthly follow up visit. Overall compliance will be the average of the monthly compliances. Subject's compliance will be recorded on the case report forms (CRFs)

2.8.4 Excluded medications and treatments

In addition to the study drugs, during the study the participants will be continuing with the HIV/AIDS standard treatments i.e. ARV drugs and prophylaxes against opportunistic infections. After being recruited into the study if it becomes clinically essential that the participant must take antiplatelets, anticoagulants and/or methotrexate then the patient will be withdrawn from the study. Over the counter ASA will not also be permitted during the period.

NSAIDs will have to be avoided wherever possible but short term intermittent NSAID use will be allowed. NSAIDs should not be co-administered with the trial treatment for more than 2 consecutive weeks. Paracetamol can be considered as an alternative analgesic and will permitted within the study. The use of co- medications will be checked at each visit and will too be recorded on the CRFs.

2.9 Subject enrolment and randomization

2.9.1 Recruitment

Subjects will be recruited from CTCs in Dar es Salaam. Patients attending CTCs at each site will be informed about the proposed study at the CTCs and will be invited to participate by the study nurses and doctors (who are also CTC staff). Prior to performing any study specific procedure (including screening procedures to determine eligibility), a signed consent form will be obtained for each subject once they have been diagnosed to be HIV- infected. The consent form will describe the purpose of the study, the procedures to be followed, and the risks and benefits of participation. The study nurse who will be part of the CTC staff and well informed of the study protocol, will conduct the informed consent discussion and will check that the subject comprehends the information provided and answer any questions about the study. Should there be an issue raised by the patients that the study nurse cannot address, the study doctor will be at hand to assist. Consent will be voluntary and free from coercion. The study nurse that will have conducted the consent discussion will also sign the informed consent form. A copy of the consent form will be given to the subject and the fact that the subject has been consented to the study will be documented in the subject’s record. Then, a screening number will be given to the already consented patient by the study nurse. The patient will be attended by the study doctor and assessed for eligibility. When the all the inclusion and exclusion criteria have been addressed and the eligibility of the subject confirmed, the subject will be consecutively recruited into the study and will be given a study number by the study doctor. The patient will go with the study number to the pharmacy and show it to the study pharmacist. The study pharmacist will give a box containing the study drugs according to the study number and the randomization schedule.

On average, 10 patients will be registered for care per week at each study site making 20 patients per week and 80 patients per month. Therefore, for 454 patients the recruitment is expected to last

for at least 6 months. Reasons for ineligibility for patients and reasons for non-participation of eligible subjects will be documented.

2.9.2 Eligibility Criteria

Patients will be assigned to a randomized study treatment only if they meet all of the inclusion criteria and none of the exclusion criteria.

2.9.2.1 Inclusion Criteria

Each patient must meet all of the following criteria to be enrolled in this study:

- Consenting newly recruited male or female HIV-infected patients
- ARV drugs naïve initiating on ARV drugs
- Aged 18 years and above
- Willing to stay in Dar es salaam for at six months
- Willing to attend HIV clinics at **Mwananyamala** or Mbagala Rangi Tatu hospitals for at least six months

2.9.2.2 Exclusion Criteria

Patients meeting any of the following criteria will be excluded from the study:

- Previous intolerance or allergy to ASA or any ASA products
- Asthmatics
- Predisposition to bleeding
- Antithrombotic therapy
- Therapy with prohibited drugs (see appendix 5)
- Active or history of peptic ulcer disease
- Pregnancy
- Severe renal disease (eGFR <30 mil/min/1.73 m^2)

2.9.3 Randomization Procedures

Randomization will be performed separately at each study site. Each study site will have 227 eligible participants. Patients will be randomly assigned to receive either 75mg ASA or a placebo in a ratio of 1:1. A statistician who will not be part of the study will prepare the randomization

schedule using block randomization of 23 blocks. The randomization schedule will contain study numbers against their respective arbitrary study letters (P and Q) representing the study treatment groups A and B. The statistician will be aware of which arbitrary study letter represents which study treatment group and hence study drug. The randomization schedule will be sent to an independent pharmacist for labeling (handwriting arbitrary study letter and month number) of the study drug boxes. The bottles containing the study drugs and the randomization schedule will then be sent to the pharmacy at the respective study sites. The schedule will be provided to the study pharmacist (recruited from CTC staff) and sealed envelopes containing the treatment allocation of each randomization code will remain with the statistician who will give it to the investigator at the time of data analysis.

2.9.4 Blinding Arrangements

This will be a double- blinded study where all members of the research team and the subjects will be blinded to treatment allocation. Only the statistician who will make the randomization schedule and the independent pharmacist who will label the study drug boxes with the arbitrary study letters will be unblinded.

2.9.5 Breaking of the Study Blind

2.9.5.1 On Study

Breaking the code will only occur upon recommendation by the Data and safety monitoring board (DSMB) during the study duration i.e. before completion of the study.

2.9.5.2 Following Completion of the Study

The study drug codes will only be available once all data collected will have been entered into the database for every participant and the data base has been finalised.

2.9.6 Subject Withdrawal

2.9.6.1 Reasons for withdrawal

The investigator may withdraw a patient from the study treatment procedures if the patient;

- Will be in violation of the protocol and/or
- Will experience an SAE that is related to the study drug and/or
- Will develop, during the course of the study, symptoms or conditions listed in the exclusion criteria and/or

- Will require a medication that is prohibited by the protocol

The investigator will also withdraw all participants from the study treatment if the study is terminated. Patients will be free to withdraw from the study at any time upon their request.

All the patients who will be withdrawn from the study will be followed up to completion of six months of follow up.

2.9.6.2 Handling of withdrawals and losses to follow- up

When a patient withdraws from the study, the reasons for withdrawal shall be recorded by the investigator on the relevant page of the CRF. Whenever possible, all patients who will withdraw from the study prematurely will continue to undergo scheduled visits for study assessments (follow-up).

Patients who will fail to return for study assessments will be contacted by the research team in an attempt to have them comply with the protocol. No contact after two documented phone calls to the patient and one to the next of kin will be considered a loss to follow up.

2.9.6.3 Replacements

There will be no replacement of patients who will discontinue the study by further recruitment to maintain the required minimum sample size.

2.9.7 Trial Closure

The trial will be closed once the last patient to be enrolled into the study has completed their last follow up visit.

2.9.8 Continuation of therapy

No study medication will be issued to a patient after the final treatment day (6 months of study medication use). Patients will continue with their HIV/ AIDS standard treatments.

2.10 Study Visits and Procedures schedule

There will be a total of 7 visits for each patient. At visit 0, patients attending CTCs at each site will be informed about the proposed study and will be invited to participate by the study nurses and doctors. Informed consent will be obtained from the patients by the study nurse who is an employee at the respective CTC. After the patient has given an informed consent, he/she will then be asked

to provide their demographic information. For all the male patients, confirmation of eligibility will be after the demographics while for female patients after the medical history they will be asked to provide urine for pregnancy test. A positive test will exclude them from the study. Once one is found eligible, they will be consecutively enrolled into the study followed by medical history and a clinical examination including blood pressure, height and weight measurements. Once the clinical examination is complete, participants will be randomized to the treatment groups and then blood samples will be taken for baseline investigations before initiation of the ARV drugs and the study drugs. The baseline investigations will include RFT, LFT, CD4 counts and FBP which are routinely taken before initiation of ART. In addition, specifically for this study, blood samples for viral load, lipid profile, plasma levels of sCD14 and sP- selectin, biomarkers of T cell activation and exhaustion will be taken at baseline.

In the subsequent monthly visits, patients will have medical history taken and clinical examination except height measurement (done only at baseline) done by the study doctor. In each of the monthly visits data on adherence to ARV drugs and compliance to study drugs, AEs, morbidity, mortality and co-medications will be collected. Additionally, patients will get their study medications refill and be counselled on adherence to ART and compliance to study drugs. In visit 0 and in all follow up visits, patients will also be asked to return to the clinics with their study drug boxes and ARV drug bottles in the next follow up visit for pill count. In addition to the aforementioned information, at months 2 and 3 blood samples for HIV viral load will be taken and also results for routine viral load test measurements for month 6 will be traced from the patients' files and/or medical records and will be included in the analysis. For CD4 count blood samples will be taken at 3 months and results for routine CD4 count measurements at month 6 will be traced from the patients' files and/or medical records and will be included in the analysis. For plasma levels of sCD14 and sP- selectin and for biomarkers of T cell activation and exhaustion blood samples will be taken at months 3 and 6. Blood samples for lipid profile, FBP, RFT and LFT will be taken only at 6 months. Urine samples will be taken from female participants for urine pregnancy test (UPT) during each monthly visit to check if they are pregnant.

	STUDY PERIOD	Screening and Randomization	Study Treatment	Follow- up			
	VISIT NUMBER	Visit 0	Visit 0	Visits 1 – 7			
	MONTH	Month 0	Month 0	Months 1-6	Month 2	Month 3	Month 6
PROCEDURES	Informed consent	X					
	Demographic information	X					
	Medical history	X		X			
	Clinical examination	X		X			
	Height measurement	X					
	Weight measurement	X		X			
	Blood pressure measurements	X		X			
	Urine pregnancy test	X		X			
	Confirm eligibility			X			
	Randomization	X					
	CD4 count	X				X	X
	Viral load	X			X	X	X
	FBP	X					X

	**RFT**	**X**					**X**
	**LFT**	**X**					**X**
**PROCEDURES**	**Lipid profile**	**X**					**X**<br>**X**
	**sCD14, sP-selectin**	**X**				**X**	**X**
	**CD38, HLA-DR, PD-1**	**X**				**X**	**X**
	**Study drug dispensing**		**X**	**X**			
	**Adherence to ART check**			**X**			
	**Compliance to ASA check**			**X**			
	**Morbidity check**			**X**			
	**Adverse event check**			**X**			
	**Mortality check**			**X**			
	**Concomitant medication check**			**X**			

Table 2.1: Schedule of Assessments at each site.

2.11 Clinical and laboratory assessments

2.11.1 Demographic information

Demographic information that will be collected will include age, sex, place of residence, marital status, level of education and employment status.

2.11.2 Medical History

A detailed medical history will be taken from the patients by the study doctor. Some of the information to be taken will include when patient diagnosed with HIV, ARV regimen to be initiated, co- medications taken and/or to be initiated and information on some risk factors for cardiovascular disease (tobacco use/ smoking, hypertension, diabetes, physical inactivity and family history of cardiovascular disease). Also, information on history of active or previous peptic ulcer disease, epigastric pain, vomiting blood, passing blood in stools or black stools will be sought at enrollment and the follow up visits. The information will be obtained from the patients themselves, their files and/or treatment charts.

2.11.3 Clinical examination

Blood pressure, height and weight measurements and a thorough examination of the respiratory system, lymphatic system, skin and mucous membranes, gastrointestinal tract (GIT), cardiovascular, central nervous system and musculoskeletal systems will be conducted by a study doctor at enrolment and at each follow up visit except for height measurement which will only be done at visit 0.

2.11.4 Weight, Height and Blood pressure measurements

Weight will be measured using an analogue scale without shoes and will be recorded to the nearest 0.5 kg. Height will be measured by a wall-mounted tape-measure with a scale up to 0.1 cm and then converted to meters. Weight in kg will then be divided by height in meters squared to obtain BMI. BMI will be categorized into groups according to World Health Organization (WHO) criteria.

Blood pressure will be measured after the respondent has rested for at least 5 minutes, using a manual sphygmomanometer. The subject's left arm will be placed at the heart level. Two measurements will be taken at an interval of five minutes between readings. Patient’s blood pressure will be the average of the two readings.

2.11.5 Urine Pregnancy Test

At screening, all the female participants will be given urine containers to provide urine.
A urine for pregnancy test will be performed using a commercial kit for analysis of a urine sample for Human Chorionic Gonadotrophin (HCG) and the results will be interpreted according to the manufacturer's instructions.

2.11.6 Laboratory tests

Laboratory tests for CD4 count at 3 months, lipid profile, FBP, RFT and LFT will be performed at the MUHAS Clinical Research laboratory (MCRL) at MUHAS. HIV viral load test at baseline, 2 and 3 months will be performed at the Central Pathology Laboratory at the Muhimbili National Hospital while HIV viral load and CD4 count at month 6 will be performed at the respective study sites laboratories. Plasma levels of sCD14, sP- selectin and biomarkers of T cell activation and exhaustion will be done in a laboratory at Kumamoto University, Japan.

- HIV Viral load testing will be performed using Abbott m 2000 rt (Applied biosystems ®, Singapore) with the lowest detection limit of 45 copies/ ml.
- Lipid profile, RFT and LFT will be performed using COBAS Integra 400 Plus (Roche Instruments Center AG, Switzerland)
- FBP will be performed using Sysmex (Sysmex Corporation, Japan)
- CD4 counts will be performed using FACS Calibur (BD Biosciences, USA)
- The pro inflammatory biomarkers profile will be analyzed using customized BDTM Cytometric Bead Array (CBA) (BD Biosciences, San Jose, California). Individual plex assay for Human P-selectin and sCD14 will be used to configure a multiplex system for quantitation of the targeted biomarkers simultaneously.
- T cell activation and exhaustion will be determined as follows: PBMCswill be thawed and stained with live and dead fixable yellow (Invitrogen, Carlsbad, CA). Then aliquots of the stained cells will be further stained with with CD3 PerCP (BioLegend, San Diego, CA), CD4 AF700, CD8 APC-H7, HLA-DR PE, CD38 APC, and PD-1 BV421 (BD Biosciences, San Jose, CA) and with CD14 Pacific Blue, CD16 PE, and CD69 PE-Cy7 (BD Biosciences, San Jose, CA) and acquired on an LSRII flow cytometer (BD Biosciences, San Jose, CA), using FacsDiva software. Percentages of activated (CD38+HLA-DR+) and exhausted (PD1+) CD4

and CD8 T cells and monocyte subsets (CD14/CD16/CD69) will be determined by gating based on isotype controls using FacsDiva software (BD Biosciences, San Jose, CA).

Performance of the various laboratory tests will be done according to the SOPs in the laboratories and interpretation of results will be in line with the instructions of the manufacturers of the respective test kits/systems.

A total of 20 ml of whole blood will be drawn from each patient in three portions. i.e. 1) 4ml in one K2 EDTA tubes for FBP and CD4 count, 2) 12mls in four K2 EDTA tubes for plasma (viral load and platelet and monocyte activation biomarkers) and PBMCs (T cell activation and exhaustion) separation and 3) 4 ml in one red topped tube for lipid profile, RFT and LFT. All sample portions will be transported in a cool box at room temperature by motorcycle to the MCRL for processing, storage and analysis. Time allowable from sample collection to processing of FBP and CD4 count is 6 hours. At the laboratory, 50 microliters will be used for FBP and 50 microliters for CD4 count.

For plasma and PBMCs separation, 12 ml sample portion will be centrifuged to separate plasma (at 1500 X g for 10 minutes at minimum acceleration and deceleration) within 2 hours from the time of sample collection. Separated plasma (approximately 6 ml) will be stored in 1.5ml and 4.5ml portions and immediately stored at -80º Celsius. 1.5ml portion will later be used for viral load testing while the 4.5 ml portion will be transported to Kumamoto University-Japan for platelet and monocyte activation biomarkers testing.

PBMCs will be separated from cellular sediment of whole blood obtained from plasma separation procedure above (see appendix 10) and then stored at -80º Celsius to be transported to Kumamoto University-Japan for T cell activation and exhaustion analysis. Lipid profile, RFT and LFT, will be tested from serum obtained from centrifugation (at 2500 revolutions per minute for 15 minutes) of the last 4 ml sample portion (in red topped tube) The time allowable from sample collection to processing is 6 hours. The obtained serum will be stored at - 80º Celsius awaiting analysis. All the frozen samples will slowly be brought to room temperature at the time of analysis.

2.11.7 Other assessments

During clinical evaluation at each monthly visit, the attending clinician will look for occurrence of any AE since the last visit and all the AEs will be recorded in a specially designed form.

Assessment of compliance to study drugs will be by pill count. Patients' remaining monthly tablets of their study drugs will be counted to ascertain compliance at each monthly follow up visit. Overall compliance will be the average of the monthly compliances.

Adherence to ART will be measured based on pill counts. Patients' will be asked to return with the ARV drug bottles every time they come to the clinic for their follow up visits. At each visit, pills remaining in bottles will be counted by the study doctor and the percentage of these pills to the dispensed pills during the previous visit will be calculated based on the dose and the number of days dispensed. The pill count adherence percent will be obtained by dividing the number of pills consumed by the total number of pills dispensed at the last visit and multiplied by 100. Overall adherence will be the average of the monthly adherences. For patients who will not last in the study for all the 6 months, their adherence to ART will be calculated by taking the average of the monthly adherences of months they have been in the study. Greater than 90% overall adherence by pill count will be considered adherence(58).

Morbidity will be defined as health care facility visits for medical attention/ care because of illness and/or the number of admissions to a health care facility/ hospital. At each monthly visit, patients will be asked if they visited any health care facility to seek medical care/ treatment and/ or if they were admitted on medical basis since last visit. The reason for visit or admission will be traced from the patient or medical and/or clinical records respectively.

All-cause mortality will be defined as confirmed death of any cause with certification of death by medical practitioner or a verbal or telephone confirmation of death from a relative or friend. Clinical conditions associated with death will be determined by verbal autopsy and/or review of clinic and hospital records. Formal autopsies will not be conducted. Conditions will be defined as associated with death if they will be diagnosed within one month prior to or at time of death or were thought to be related to death after review of the medical records. More than one diagnosis will be possible for each death.

2.12 Adverse event reporting

2.12.1 Definitions

AE: Any untoward medical occurrence in a patient enrolled into this study regardless of its causal relationship to study treatment.

SAE

An SAE is defined as any AE that: results in death; or is immediately life threatening; or requires inpatient hospitalization; or requires prolongation of existing hospitalization; or results in persistent or significant disability/incapacity; or is a congenital anomaly/birth defect.

Important medical events will be considered an SAE when, based upon appropriate medical judgement, they may jeopardize the patient and may require medical or surgical intervention to prevent one of the outcomes listed in this definition.

Non-SAE

A non-SAE is defined as any AE that: does not result in death; or is not immediately life threatening; or does not require inpatient hospitalization; or require prolongation of existing hospitalization; or does not result in persistent or significant disability/incapacity; or is not a congenital anomaly/birth defect.

SUSAR: A SUSAR is any SAE that is both suspected to be related to the study treatment and is unexpected (i.e. not consistent with applicable product information).

2.12.2 Assessment and Documentation of Adverse Events

For the purposes of this study the investigator is responsible for recording all AEs, regardless of their relationship to study drug, with the following exceptions:

- Conditions that are present at screening and do not deteriorate will not be considered adverse events.
- Abnormal laboratory values will not be considered adverse events unless deemed clinically significant using local laboratory reference ranges(59) and documented as such.

The description of each AE on the CRF will include: A description of the AE; The onset date, duration, date of resolution; Severity (mild, moderate or severe); Seriousness (i.e. is it an SAE?); Any action taken, (e.g. treatment, follow-up tests); The outcome (recovery, death, continuing, worsening); The likelihood of the relationship of the AE to the study treatment (Unrelated, Possible, Probable, Definite).

The severity and relationship of an AE will be assessed as appendix attached. The seriousness of an AE will be assessed by an investigator according to the definition of an SAE with the following exception:

- Hospitalization due to progression of disease will be considered an SAE.

Changes in the severity of an AE will be reported. AEs characterized as intermittent will be documented for each episode. All AEs will be followed to adequate resolution, where possible.

2.12.3 Eliciting Adverse Event Information

Adverse events will be recorded from the time the patient signs the informed consent form until 30 days after the last dose of study medication. At every study visit patients will be asked "How have you felt since your last visit?" in order to elicit any medically related changes in their well-being. They will also be asked if they have been hospitalized, had any accidents, used any new medication or changed concomitant medication regimens. In addition, AEs will be documented from physical examination findings, clinically significant laboratory results or other documents (including correspondence from primary care physicians) that are relevant to patient safety.

2.12.4 Serious Adverse Event Reporting

2.12.4.1 SAEs

Any SAE occurring in a study participant will be reported to the MUHAS and the National ethical committees within 24-72 hours of occurrence. The safety reporting forms will be completed, signed and submitted by an investigator.

2.12.4.2 SUSARs

All SUSARs occurring in a study participant will be reported in an expedited fashion (i.e. within 15 calendar days of first knowledge), or for fatal or life-threatening events, an initial or full report within 7 calendar days and a follow-up report if necessary, within the 15-calendar day timeframe. An investigator will complete, sign and submit the SUSAR report."

2.13 Statistical Methods

2.13.1 Sample Size estimation

The required sample size is 227 participants in each treatment arm making a total of 454 participants.

Assuming that the proportion of patients with < 50 copies/ml at 8 weeks in the control group (ARV alone) is 75%(60), then 227 patients will be required, in each study treatment group, if a relative risk for the ARV + ASA treatment group is 1.15, assuming loss to follow up of 10%, a power of 80% and a significance level of 0.05.

Item	Value
Proportion of patients with < 50 copies/mL at week 8 after ART initiation in the control group (ARV alone)	75%
Relative risk =1.15 for intervention group(ARV + ASA)	1.15 (i.e. 86% in intervention group(ARV + ASA))
Expected loss to follow up	10%
Significance level for a two-sided test	0.05
Power	80%
Total patients in each group	227

2.13.2 Population to be analyzed

The subject population whose data will be subjected to the study to analyses is the intention to treat population.

2.13.3 Statistical Analysis Plan

The data will be double entered into a computer by two different data entry clerksand the entered data will then be verified and cleaned before being subjected to analysis using the SPSS statistical program. Intention to treat analysis will be done.

Chi- square or Fisher’s exact test will be used for comparison of proportions and Student’s t test for comparison of means between the two treatment groups.

Descriptive statistics will also be used where appropriate. A two tailed P-value of <0.05 will be considered significant.

2.14 Data Management

2.14.1 Data collection and storage

Data will be collected through interviewing the patients, from their medical files/treatment charts and source data documents and will be recorded on the CRF. The CRFs will be checked for completeness and accuracy against the source data. Original CRFs will be used when entering information into the computer database. The database will be checked against the CRFs for accuracy. Data analysis will begin after ensuring that all the data have been accurately entered.

Data will be kept in a password secured computer to ensure confidentiality and all the blood samples and documents during the study period will be stored by the investigator until time for destruction after the data have been analyzed and published.

2.15 Ethical considerations and approval

2.15.1 Ethical issues

The main aspect of this trial that raises ethical issues is the fact that it is necessary to collect samples for HIV-related laboratory testing. During follow up the patients may be asked to share information about their wellbeing and some of the information may be viewed by them as sensitive. Subject confidentiality will therefore be strictly held in trust by the participating investigators and the research staff. This confidentiality will be extended to cover testing of biological samples in addition to the clinical information relating to participating subjects. The study protocol, documentation, data and all other information generated will be held in strict confidence. No information concerning the study or the data will be released to any unauthorized third party, without prior written approval. All laboratory specimens, evaluation forms, reports and other records that will leave the site will be identified only by the subject study number to maintain subject confidentiality. Clinical information will not be released without written permission of the subject, except as necessary for monitoring by the ethical committees.

If bleeding occurs, study drug will be stopped and immediate clinical assessment will be undertaken and the participant will be managed accordingly.

2.15.2 Ethical approval

The study will be conducted according to ICH GCP guidelines and the Declaration of Helsinki (Version 2008), and it is the responsibility of the Investigators to ensure adherence. The investigator will ensure all personnel involved in the study are already trained for Good Clinical Practice (GCP) and/or Good Clinical and Laboratory Practice (GCLP). Study personnel will also be trained on standard operating procedures (SOPs) that are related to the protocol and will follow the GCP and GCLP guidelines.

Patients who will present with SAE that will be related to the study drugs will be stopped from taking the study drugs and referred to the study clinician for management. These patients will then be followed up to complete the 12 months of follow up.

This protocol will be submitted concurrently to the institutional ethical committees of the Muhimbili University of Health and Allied Sciences (MUHAS) and the National Health Research Ethics Committee at the National Institute of Medical Research (NIMR) for ethical approval. Letters of protocol approval by the ethical committees will have to be obtained prior to the commencement of the study, as well as approval for other study documents subject to ethical committees' review. Approval of the protocol and the study drugs will be done by the Tanzania Medicine and Medical Devices Authority (TMDA).

The Investigator and the Supervisors will also be responsible for informing the ethics committees of any SAEs as required, and submitting six monthly report as required. No study materials will be obtained from study participants before approval from the relevant bodies.

The investigator and statistician will be responsible for data analysis. Staff will also be responsible for coordinating the response to any SAE's that arise during the course of the trial and reporting these if indicated to the investigator, who will then report to the appropriate board/committee. Continuous study monitoring will be performed by the investigator in collaboration with an internal monitor who will closely follow up and review study progress, adverse events, any protocol deviations, laboratory data and clinical information. The data and safety monitoring board (DSMB) will also independently monitor the study at intervals to review progress reports, safety data, trial performance and progress and make pertinent recommendations to research ethics committees, regulatory authority, directorate of research and publications at MUHAS and the investigator. The DSMB will comprise of a clinical pharmacologist, a physician and a biostatistician. The DSMB will hold scheduled meetings prior to commencement of data

collection, at 3 months after data collection has started and at the end of the study. These meetings will be headed by the clinical pharmacologist who will be the chairperson of the DSMB.

2.15.3 Modifications of the protocol

This study will be conducted in compliance with the current version of the protocol. Any change to the protocol document or Informed Consent Form that affects the scientific intent, study design, patient safety, or may affect a participant's willingness to continue participation in the study will be considered an amendment, and therefore will be written and filed as an amendment to this protocol and/or informed consent form. All such amendments will be submitted to the ethical committees, for approval prior to becoming effective

2.15.4 Protocol Deviations

All protocol deviations will have to be recorded in the patient record on the CRF and will have to be reported to the investigator. Protocol deviations will be assessed for significance by the investigator. Those deviations that will be deemed to have a potential impact on the integrity of the study results, patient safety or the ethical acceptability of the trial will be reported to the ethical committees. Where deviations to the protocol will identify issues for protocol review, the protocol will be amended.

2.15.5 Participant Reimbursement

Eight thousand Tanzanian shillings (8000 Tshs) will be given to every participant to cover for transport cost and lunch whenever a patient will have to come for follow up visits other than his/her routine care schedule.

2.15.6 Study Insurance

The investigator will purchase an insurance coverage from the National Insurance Corporation for the proposed study. This insurance will be payable at an event of death of the life insured and/ or total permanent disability resulting from the study drugs but not from any other cause which has nothing to do with the study.

2.16 Study limitations and mitigation measures

Adherence to both ARV drugs and ASA measured by pill count may not be fully objective as patients may throw away their pills or forget to return with their study drug boxes and bottles to

the follow up visits. To ensure that adherence by pill count, which will be used in the proposed study, reflects the nearly true adherence, both at consenting and throughout the study, the participants will be reminded by a text message a day before clinic visit to carry their drug boxes and bottles with them on the visit day. The participants will also be educated on the importance of complying with the study procedure including adherently taking their study drugs and faithfully returning their study drug boxes and bottles for pill count. Ideally, measuring drug levels would be the most appropriate method of assessing adherence. However, the method is very costly and cannot be accommodated within the available limited budget.

Since some of the laboratory results will rely on the information available in the clinic records, some laboratory results may be missing. Special efforts will be made to emphasize the importance of performing the follow up laboratory tests as recommended in the care and treatment guideline. Such efforts include

- The research team will be properly trained on the protocol with the emphasis on time points where blood samples must be routinely collected (to ensure that they are collected) and recorded for the study
- There will also be flow charts in the blood sample collection rooms showing time points to collect samples
- Posters will also be put on the tables of the study doctors indicating when these investigations should be done

2.17 Dissemination plan

The study findings will be disseminated by:

- Publications in peer review journals
- Presentation at International, regional and local scientific conferences
- Public defense of the thesis at University level
- Presentation of the study reports to the MoHCDGEC/policy makers, the National Aids Control Programme, NIMR etc.
- Official meeting with the study participants.

3. BUDGET AND ITS JUSTIFICATION

3.1 Budget

Category	Item Description	Specification	Quantity per	Quantity	Unit cost(Tshs) Per item	Total cost(Tshs)
		**Tests**	**kits**	**sample size**		
**Laboratory tests**	HIV Viral load	3		908	45,000.00	40,860,000.00
	CD4 count	2		908	15,000.00	13,620,000.00
	Lipid profile	2		908	6,400.00	5,811,200.00
	sCD14	3	8	1362	-	-
	sP- selectin	3	15	1362	-	-
	UPT	2722	23	303	12,000.00	276,000.00
	FBP	2		908	16,000.00	14,528,000.00
	RFT	2		908	2,860.00	2,596,880.00
	LFT	2		908	3,280.00	2, 978,240.00
	**Sub Total**					**80,670,320.00**
**Equipment**	BP machine			2	150,000.00	300,000.00
	**Sub Total**					**300,000.00**
		**Tablet**	**days**	**sample size**		
**Study drugs**	Placebo	100000	183	227	81.00	8,100,000.00
	75 mg ASA	50000	183	227	81.00	4,050,000.00
	**Sub Total**					**12,150,000.00**
		**staff**	**remuneration per patient for each staff**	**visits**	**remuneration per 6 months for all staff**	
**Personnel**	Pharmacists	2	500.00	7	1,589,000.00	1,589,000.00
	Prescreening nurse	2	500.00	7	1,589,000.00	1,589,000.00
	Phlebotomy nurse	2	500.00	7	1,589,000.00	1,589,000.00
	Informed consent nurse	2	1,000.00	7	3,178,000.00	3,178,000.00
	Doctor	2	1,000.00	7	3,178,000.00	3,178,000.00
	Investigator	1	N/A	N/A	1,800,000.00	1,800,000.00

	**Sub Total**					**12,923,000.00**
			**visits**	**sample size**		
**Direct Cost**	Ethical clearance (NIMR)				1,100,000.00	1,100,000.00
	Ethical clearance (MUHAS)				1,000,000.00	1,000,000.00
	Ethical clearance (TMDA)				-	-
	Clinical trial participation insurance	454	7	454	12,841.60	5,830,086.40
	Voucher for telephone tracing				309,545.00	309,545.00
	Sample transport				4,952,730.00	4,952,730.00
	Stationeries				854,345.00	854,345.00
	Public transport fare and lunch	454	1	454	8,000.00	3,632,000.00
						**17,678,706.40**
	**TOTAL BUDGET**					**123,722,026.40**

3.2 Budget justification

The proposed study is supported by HIV Implementation Science Research Project and Transforming Health Professions Education in Tanzania (THET) Project, in addition application for possibility of partial support, especially from the Sida small grants, under the Sida-MUHAS collaboration, are ongoing. The estimated 123,722,026.40 Tshs will be required to carry out this study.

About 80,670,320.00 Tshs of the budget has been allocated as cost for laboratory tests (including the consumables and reagents) which will be carried at different points during the study period. A total of 300,000 Tshs will be spent on buying equipment for measuring blood pressure at the two study sites.

Each participant will need a monthly supply of study drugs for a period of six months (183 tablets for each participant) and this is estimated to cost 12,150,000.00 Tshs.

The stipend for the PhD will be at a rate of 300,000.00Tshs per month. The PhD student will visit the study sites every day to recruit participants for the study, ensure that the SOPs for blood sample collection and storage are followed and to collect samples from the sites for transportation to MUHAS laboratory and CPL for analysis. This will be an ongoing activity for about one year.

Samples will be transported by motorcycle from the study sites to the MUHAS laboratory and CPL at an estimated cost of 50000.00 Tshs per day hence amounting to 4,952,730.00 Tshs for the whole study period. Since this is a follow up study, continuous visits, participant enrollment and collection of blood sample will be required. Therefore, the study will recruit research assistants (doctors, pharmacists and nurses working at the study sites). It is essential to have these assistants to save time and ensure that there is continuous recruitment and sample collection. The allowances of each assistant will be paid (per patient) monthly for the whole study period. Telephone calls will be used to remind patients of their next follow up visits and to trace those who will have not shown up for their visits. This is estimated to cost 309,545.00 Tshs for the whole study period. A cost of 854,345.00 Tshs is estimated to be used for stationeries and photocopying of the consent forms and the page CRFs. The cost of photocopying a page is 100.00 Tshs.

All the participants are going to be insured during the one year of treatment and follow up in line with TMDA clinical trials regulations. The cost for insurance is 12,841.00 Tshs per patient for one year. It is not expected that the participants will come for follow up visit that is not in their routine CTC visits therefore only 1 unscheduled visit is anticipated. The study will have to provide 8,000 Tshs to every participant for public transport fare and lunch should there be a visit other than their routine clinic visits.

4. WORK PLAN

ACTIVITY	May 2018	Jun-July 2018	Aug-Nov 2018	Dec 2018-May 2019	Jun-Nov 2019	Dec 2019-May 2020	Jun-Nov 2020	Dec 2020-May 2021	Jun-Nov 2021	Dec 2021-May 2022
Preparing final full research proposal.										
Presenting the final full research proposal to the departmental seminar for review.										
Site previsit and finalization of the proposal.										
Submission of the proposal for full registration and ethical clearance.										
Attendance of short courses.										
Recruitment of research assistants and training them on the research protocol.										
Recruitment of the participants,data collection and follow up.										
Preparation of 1st and 2nd manuscript for publication.										
Presentation of research findings at the										

national and international scientific conference										
Preparation of the 3rd and 4th manuscript for publication.										
Preparation of the dissertation, submission and defence.										

5. REFERENCES

1. Joint United Nations Program on HIV/AIDS(UNAIDS). UNAIDS Data 2017. 2017.
2. Frank J. Palella, Jr. M, Rose K. Baker M, Anne C. Moorman, BSN M, Joan S. Chmiel P, Kathleen C. Wood B, John T. Brooks M, et al. Mortality in the Highly Active Antiretroviral Therapy Era; Changing Causes of Death and Diseases in the HIV Outpatient Study. J Acquir Immune Defic Syndr. 2006;43(1):27–34.
3. Hogg R, V L, JA S, S G, M B, M B, et al. Life expectancy of individuals on combination antiretroviral therapy in high-income countries: a collaborative analysis of 14 cohort studies. Lancet. 2008;372(9635):293–9.
4. Group he DC on AE of A-H drugs (D:A:D) S. Factors associated with specific causes of death amongst HIV-positive individuals in the D : A : D study. AIDS. 2010;24(10):1537–48.
5. Sackoff JE, Hanna DB, Pfeiffer MR, Torian L V. Causes of Death among Persons with AIDS in the Era of Highly Active Antiretroviral Therapy : New York City. Ann Intern Med. 2006;145(6):397–406.
6. The Antiretroviral Therapy Cohort Collaboration. Causes of Death in HIV-1–Infected Patients Treated with Antiretroviral Therapy, 1996–2006: Collaborative Analysis of 13 HIV Cohort Studies. Clin Infect Dis. 2010;50(10):1387–96.
7. Rodger AJ, Lodwick R, Schechter M, Deeks S, Amin J, Gilson R, et al. Mortality in well controlled HIV in the continuous antiretroviral therapy arms of the SMART and ESPRIT trials compared with the general population. AIDS. 2013;27(6):973–9.
8. Marin B, Thiébaut R, Bucher HC. Non-AIDS-defining deaths and immunodeficiency in the era of combination antiretroviral therapy. AIDS. 2009;23(13):1743–53.
9. Islam FM, Wu J, Jansson J, Wilson DP. Relative risk of cardiovascular disease among people living with HIV : a systematic review and meta-analysis. HIV Med. 2012;13:453–68.
10. Freiberg MS, Chang CH, Kuller LH, Goetz MB, Leaf D, Oursler KA, et al. HIV Infection and the Risk of Acute Myocardial Infarction Matthew. JAMA Intern Med. 2013;173(8):614–22.
11. Hemkens LG, Bucher HC. Novel therapeutic concepts HIV infection and cardiovascular disease. Eur Heart J. 2014;35:1373–81.

12. Lichtenstein KA, Armon C, Buchacz K, Chmiel JS, Buckner K, Tedaldi EM, et al. Low CD4 + T Cell Count Is a Risk Factor for Cardiovascular Disease Events in the HIV Outpatient Study. Clin Infect Dis. 2010;51(4):435–47.
13. Lang S, Mary-krause M, Simon A, Partisani M, Gilquin J, Cotte L, et al. Replication and Immune Status Are Independent Predictors of the Risk of Myocardial Infarction in HIV-Infected Individuals. Clin Infect Dis. 2012;55(4):600–7.
14. Hunt PW, Brenchley J, Sinclair E, Mccune JM, Page-shafer K, Hsue P, et al. Relationship between T Cell Activation and CD4+ T Cell Count in HIV-Seropositive Individuals with Undetectable Plasma HIV RNA Levels in the Absence of Therapy. J Infect Dis. 2008;197(March 2007):126–33.
15. Hunt PW, Martin JN, Sinclair E, Bredt B, Hagos E, Lampiris H, et al. T Cell Activation Is Associated with Lower CD4 $^{+}$ T Cell Gains in Human Immunodeficiency Virus–Infected Patients with Sustained Viral Suppression during Antiretroviral Therapy. J Infect Dis [Internet]. 2003;187(10):1534–43. Available from: https://academic.oup.com/jid/article-lookup/doi/10.1086/374786
16. Brien MO, Montenont E, Hu L, Nardi MA. Aspirin Attenuates Platelet Activation and Immune Activation in HIV-1-Infected Subjects on Antiretroviral Therapy: A Pilot Study. J Acquir Immune Defic Syndr. 2014;63(3):280–8.
17. Longenecker CT, Funderburg NT, Jiang Y, Debanne S, Storer N, Labbato DE, et al. Markers of inflammation and CD8 T-cell activation, but not monocyte activation, are associated with subclinical carotid artery disease in HIV-infected individuals. HIV Med. 2013;14(6):385–90.
18. Burdo TH, Lo J, Abbara S, Wei J, Delelys ME, Preffer F, et al. Soluble CD163 , a Novel Marker of Activated Macrophages , Is Elevated and Associated With Noncalcified Coronary Plaque in HIV-Infected Patients. J Infect Dis. 2011;204:1227–36.
19. Zanni M V, Hoffmann U, Kenneth C. Arterial inflammation in patients with HIV. JAMA. 2013;308(4):379–86.
20. Kelesidis T, Kendall MA, Yang OO, Hodis HN, Currier JS. Biomarkers of Microbial Translocation and Macrophage Activation : Association With Progression of Subclinical Atherosclerosis in HIV-1 Infection. J Infect Dis. 2012;206:1558–67.
21. Poesen R, Ramezani A, Claes K, Augustijns P, Kuypers D, Barrows IR. Associations of

Soluble CD14 and Endotoxin with Mortality , Cardiovascular Disease , and Progression of Kidney Disease among Patients with CKD. Clin J Am Soc Nephrol. 2015;10(9):1525–33.

22. Reiner AP, Lange EM, Jenny NS, Ellis J, Tracy RP. Soluble CD14: genome-wide association analysis and relationship to cardiovascular risk and mortality in the older adults. Arter Thromb Vasc Biol. 2014;33(1):1–18.

23. Longenecker CT. Soluble CD14 is independently associated with coronary calcification and extent of subclinical vascular disease in treated HIV infection. AIDS. 2014;28(7):969–77.

24. Hattab S, Guiguet M, Carcelain G, Fourati S, Guihot A, Autran B, et al. Soluble biomarkers of immune activation and inflammation in HIV infection : impact of 2 years of effective first-line combination antiretroviral therapy. HIV Med. 2015;16:553–62.

25. Malherbe G, Steel HC, Cassol S, Oliveira T De, Seebregts CJ, Anderson R, et al. Circulating Biomarkers of Immune Activation Distinguish Viral Suppression from Nonsuppression in HAART-Treated Patients with Advanced HIV-1 Subtype C Infection. Mediators Inflamm. 2014;2014.

26. Wada NI, Jacobson LP, Margolick JB, Breen EC, Macatangay B, Penugonda S, et al. The effect of HAART- induced HIV suppression on circulating markers of inflammation and immune activation. AIDS. 2015;29(4):463–71.

27. Halloran JAO, Dunne E, Gurwith MMP, Lambert JS, Sheehan GJ, Feeney ER, et al. The effect of initiation of antiretroviral therapy on monocyte , endothelial and platelet function in HIV-1 infection. HIV Med. 2015;16:608–19.

28. Andre L, Mu F, Solum NO, Brosstad F, Land Q, Aukrust PAL. Enhanced activation of platelets with abnormal release of RANTES in human immunodeficiency virus type 1 infection. FASEB J. 1998;12(1):79–89.

29. Ridker PM, Buring JE, Rifai N. Soluble P- Selectin and the Risk of Future Cardiovascular Events. Circulation. 2001;103:491–5.

30. Blann AD, Nadar SK, Lip GYH. The adhesion molecule P-selectin and cardiovascular disease. Eur Heart J. 2003;24:2166–79.

31. Scialla JJ, Plantinga C, Kao WHL, Jaar B, Powe NR, Parekh S. Soluble P-Selectin Levels Are Associated with Cardiovascular Mortality and Sudden Cardiac Death in Male Dialysis Patients. Am J Nephrol. 2011;33:224–30.

32. Wolf K, Tsakiris DA, Weber R, Erb P, Battegay M. Antiretroviral Therapy Reduces Markers of Endothelial and Coagulation Activation in Patients Infected with Human Immunodeficiency Virus Type 1. J Infect Dis. 2002;185:456–62.
33. Brien MPO, Hunt PW, Kitch DW, Klingman K, Stein JH, Funderburg NT, et al. A Randomized Placebo Controlled Trial of Aspirin Effects on Immune Activation in Chronically Human Immunodeficiency Virus-Infected Adults on Virologically Suppressive Antiretroviral Therapy. Open Forum Infect Dis. 2017;1:1–10.
34. Alfonso L, Ai G, Spitale RC, Bhat GJ. Molecular targets of aspirin and cancer prevention. Br J Cancer. 2014;111:61–7.
35. Stanczuk GA, Thomsen M, Soerensen AM SE. Acetyl salicylic acid (aspirin), micronutrients and chloroquine in the management of the Acquired Immunodeficiency Syndrome (AIDS). Cent Afr J Med. 2002;48(3/4):42–9.
36. MA D, H A, NO A, O O, IA A, OA O, et al. Selenium and aspirin in people living with HIV and AIDS in Nigeria. Niger Postgr Med J. 2008;15(4):215–8.
37. THOMSEN Marianne, SIBANDA EN SG. Acetyl salicylic acid (ASPIRIN) increases the CD4+ T lymphocytes and suppresses TNF-a in HIV-I infected patients: Results of a 12 month, three-arm, placebo-controlled pilot study. AIDS vaccines Relat Top. 2004;(1):179–90.
38. Huang ES, Strate LL, Ho WW, Lee SS, Chan AT. Long-term use of aspirin and the risk of gastrointestinal bleeding. Am J Med [Internet]. 2011;124(5):426–33. Available from: http://www.ncbi.nlm.nih.gov/pubmed/21531232%5Cnhttp://www.pubmedcentral.nih.gov/articlerender.fcgi?artid=PMC3086018
39. Bangsberg DR. Less Than 95 % Adherence to Nonnucleoside Reverse-Transcriptase Inhibitor Therapy Can Lead to Viral Suppression. Clin Infect Dis. 2006;43:939–41.
40. Machado CJ, Drew M, Guimarães C. Monitoring adherence to antiretroviral treatment in Brazil : an urgent challenge. Cad Saúde Pública. 2011;27(1):67–78.
41. Paterson DL, Swindells S, Mohr J, Brester M, Vergis EN. Adherence to Protease Inhibitor Therapy and Outcomes in Patients with HIV Infection. Ann Intern Med. 2000;133(1):21–30.
42. Hogg RS, Heath K, Bangsberg D, Yip B, Press N, Shaughnessy MVO, et al. Intermittent use of triple-combination therapy is predictive of mortality at baseline and after 1 year of.

AIDS. 2002;16(7):1051–8.

43. Harrigan PR, Hogg RS, Dong WWY, Yip B, Wynhoven B, Woodward J, et al. Predictors of HIV Drug-Resistance Mutations in a Large Antiretroviral-Naive Cohort Initiating Triple Antiretroviral Therapy. J Infect Dis. 2005;191:339–47.
44. Fletcher C V, Testa MA, Brundage RC, Chesney MA, Haubrich R, Acosta EP, et al. Four Measures of Antiretroviral Medication Adherence and Virologic Response in AIDS Clinical Trials Group Study 359. J Acquir Immune Defic Syndr. 2005;40(3):301–6.
45. Miller L, Hays R. Measuring Adherence to Antiretroviral Medications in Clinical Trials. HIV Clin Trials. 2000;1(1):36–46.
46. Magnano M, Lio S, Carbini R, Germano P, Guidotti G, Mancinelli S, et al. Evaluating Adherence to Highly Active Antiretroviral Therapy with Use of Pill Counts and Viral Load Measurement in the Drug Resources Enhancement against AIDS and Malnutrition Program in Mozambique. Clin Infect Dis. 2008;46:1609–16.
47. Gedela K, Vibhuti M, Pozniak A, Ward B, Boffito M. Pharmacological management of cardiovascular conditions and diabetes in older adults with HIV infection. HIV Med. 2014;450(15):257–68.
48. Cantudo-cuenca MR. Concurrent Use of Comedications Reduces Adherence to Antiretroviral Therapy Among HIV- Infected Patients. J Manag Care Pharm. 2014;20(8):844–50.
49. Maggiolo F, Ravasio L, Ripamonti D, Gregis G, Quinzan G, Arici C, et al. Similar Adherence Rates Favor Different Virologic Outcomes for Patients Treated with Nonnucleoside Analogues or Protease Inhibitors. Clin Infect Dis. 2005;40(1):158–63.
50. Yun LWH, Maravi M, Kobayashi JS, Barton PL, Davidson AJ. Antidepressant Treatment Improves Adherence to Antiretroviral Therapy Among Depressed HIV- Infected Patients. J Acquir Immune Defic Syndr. 2005;38(4):432–8.
51. Horberg MA, Silverberg MJ, Hurley LB, Towner WJ, Klein DB, Bersoff-matcha S, et al. Effects of Depression and Selective Serotonin Reuptake Inhibitor Use on Adherence to Highly Active Antiretroviral Therapy and on Clinical Outcomes in HIV-Infected Patients. J Acquir Immune Defic Syndr. 2008;47(3):384–90.
52. Glass TR, Battegay M, Cavassini M, Geest S De, Furrer H, Vernazza PL, et al. Longitudinal Analysis of Patterns and Predictors of Changes in Self-Reported Adherence

to Antiretroviral Therapy : Swiss HIV Cohort Study. J Acquir Immune Defic Syndr. 2010;54(2):197–203.

53. Services H, Permanente K, Whitlock EP, Williams SB, Burda BU, Feightner A, et al. Aspirin Use in Adults : Cancer , All-Cause Mortality , and Harms. 2012;(132).

54. Ittaman S V, Vanwormer JJ, Rezkalla SH. The Role of Aspirin in the Prevention of Cardiovascular Disease. Clin Med Res. 2014;12(3–4):147–54.

55. DECEMBER 2017 TANZANIA HIV IMPACT SURVEY (THIS).

56. Drake BJ, Sarah D, Tina C, Troy SB. CD4 Count Recovery After Antiretroviral Therapy Initiation in Patients Infected with the Human Immunodeficiency Virus. Am J Med Sci [Internet]. 2016; Available from: http://dx.doi.org/10.1016/j.amjms.2016.05.032

57. Soomro H, Aleem S, Hussain M, Alam A, Qadeer A, Saand AR, et al. Frequency and Predictors of Non-Compliance to Aspirin Therapy in post Myocardial Infarction Patients. Glob J Health Sci. 2017;9(1):217–23.

58. Sangeda RZ, Mosha F, Prosperi M, Aboud S, Vercauteren J, Camacho RJ, et al. Pharmacy refill adherence outperforms self-reported methods in predicting HIV therapy outcome in resource-limited settings. BMC Public Health. 2014;14(1035):1–11.

59. Saathoff E, Schneider P, Kleinfeldt V, Geis S, Haule D, Maboko L, et al. Laboratory reference values for healthy adults from southern Tanzania. Trop Med Int Heal. 2008;13(5):612–25.

60. Walmsley SL, Antela A, Clumeck N, Duiculescu D, Eberhard A, Gutieŕrez F, et al. Dolutegravir plus Abacavir-Lamivudine for the treatment of HIV-1 infection. N Engl J Med. 2013;369(19):1807–18.

6. APPENDICES

6.1 Appendix 1- Informed Consent Forms

6.1.1 Informed consent form

"**THE EFFECT OF ASPIRIN ON HUMAN IMMUNODEFICIENCY VIRUS (HIV) DISEASE PROGRESSION AMONG HIV- INFECTED INDIVIDUALS INITIATING ANTI- RETROVIRAL THERAPY**."

NAME OF INVESTIGATOR: TOSI MICHAEL MWAKYANDILE

FUNDER: HIS (HIV IMPLEMENTATION SCIENCE) AND THET

ADDRESS: MUHIMBILI UNIVERSITY OF HEALTH AND ALLIED SCIENCES

P.O BOX 65001,

DAR-ES SALAAM.

Identification number: ________________________

THE INVESTIGATOR

The proposed study with the title above will be conducted by TOSI MICHAEL MWAKYANDILE, a PhD student in the department of Clinical Pharmacology at MUHAS under the supervision of Prof. E. F. Lyamuya (MUHAS) and Dr. G. A. Shayo (MUHAS).

PURPOSE OF STUDY

HIV infection continues to be a public health problem especially in African countries including Tanzania. HIV medications (ARV drugs) have improved the number of years of people living with HIV/ AIDS (PLHA) live as compared to before their introduction. Consequently, the number of complications not usually associated with HIV/AIDS such as non- AIDS defining cancers, liver, lung and heart diseases have increased. There is evidence to show that PLHA are more likely to suffer from heart disease than those who are not. Activation of one of the types of cells in our blood known as platelets and the immune system (system of the body involved in protecting the body) are said to be big contributors to the increased chance of getting heart disease among PLHA. The use of ARV drugs increases the number of white blood cells, which the HIV attacks, (CD4)

and decreases the number of HIV in the blood (HIV load). In addition, the frequency with which opportunistic infections occur is reduced by the use of ARV drugs. However, the use of ARV drugs does not completely shut down the elevated immune system activation. Due to this drawback of ARV drugs on immune activation, an additional drug is necessary for effective treatment of HIV infection.

Aspirin or Acetyl Salicylic Acid (ASA), a drug which controls inflammation and prevents platelet activation, has been shown to reduce both platelet and immune activation in HIV- infected people. Additionally, in the HIV-infected patients ASA has been shown to increase CD4 count and decreasing the number of HIV in the blood. However, all these effects have been revealed following short observation time in small number of participants. The proposed study in which you are being invited to participate is going to look at the long term benefits of ASA on the frequency of admission to the hospital and/or hospital sick visits, death due to any cause and how fast HIV disease develops to advanced stages. It is believed that the findings from the proposed study will contribute to reductions in the frequency of hospital admission and/or hospital sick visits and thus improved quality of life and survival both for the study participants and future patients.

We are also interested on looking at the effect of adding an extra medication to the usual ARV drugs on how well patients take their ARV drugs as prescribed.

WHO WILL BE IN THE STUDY

Adult male and female patients who have been found to have HIV now or those known to have HIV before but never used ARV drugs, who are attending Care and Treatment Center (CTCs) at Mbagala Rangi Tatu and **Mwananyamala** are to be started on ARV drugs are being invited. Those who are asthmatic, pregnant, have or have ever had peptic ulcer disease, have history of easy and prolonged bleeding and/or on medications affecting the ability of the blood to clot (antithrombotic therapy) will be excluded. Those who are known to be allergic to ASA or any products containing ASA will also not be included in the proposed study.

WHAT PARTICIPATION INVOLVES

If you agree to participate in this study, you will be asked questions about your health and about your background; we will also measure your height, weight and blood pressure. Blood samples will be drawn at the follow up visits during your participation.

Procedures at clinic visits

When enrolled in this study you will have **7** study-specific clinic visits; firstly, there will be checking if you qualify to participate into the study and enrollment into the study then a monthly follow up visit for six months. After agreeing to participate in the study, you will be assessed for eligibility and if you are eligible, you will be assigned by chance to either of the two treatment groups A and B. This ensures that your assignment to whichever treatment group will be purely by chance alone. During the follow up period, participants in both the treatment groups will be on ARV drugs but in group A and B participants will additionally take 75 mg ASA and a preparation with no pharmacological effect (placebo) daily in the evenings, respectively. While in the study you will be required to come to clinic every month for planned study visits. At each of these study visits, you will get your drug refills, questions regarding your health will be asked, weight will be measured and blood samples will be collected, during some of the visits, to check your health while on treatment. The samples will be sent to the laboratories at MUHAS, MNH and Japan for analysis. You will be informed about your previous laboratory results at each monthly visit. After six months of the study, you will stop treatment with the study medication but you will continue with your ARV drugs as usual at your CTC.

BENEFITS OF THE STUDY

There will be no direct material benefits to participation in this study. However, your participation will help us find out if long term use of ASA leads to slowed down speed at which HIV disease progresses to advanced stages, reductions in the frequency of hospital admission and/or hospital visits and death due any cause and thus improved quality of life and survival both for the study participants and future patients.

The proposed study will also find out the effect of adding an extra medication to the usual ARV drugs on how well patients take their ARV drugs as prescribed.

POTENTIAL RISKS OF THE STUDY

We do not anticipate that there will be serious unwanted or harmful effects with the use of added study medications. However, there may be small risk for unwanted or harmful effects involved with low dose of ASA as compared to the high doses. As with any drug, there is a small risk that you may experience some adverse effects associated with the use of ASA. Majority of such effects

are non-serious, but we will be monitoring all these events for your safety and ask you to report any symptoms or concerns during the monthly clinic visits or to contact the research staff.

You may briefly feel a small amount of pain at the needle site when we draw blood from you, and possibly have some bruising or swelling which will disappear in few days. The volume of blood collected is small **(**20mls**)** and more or less the same as that normally collected if you were not part of the study and it is not expected to affect your health negatively.

FREEDOM TO PARTICIPATE IN THE STUDY

We would like to stress that your participation in this study is strictly voluntary. Should you decide not to participate; it will not affect the treatment or management that you will receive from the hospital. You are also free to withdraw your participation in this study at any point. If it is your wish for us to destroy any stored samples we have collected from you, we will do so. Any such decision will be respected and will not influence the quality of health care that you will receive here at the CTC or elsewhere.

PAYMENT TO PARTICIPATE IN THE STUDY

There are no payments for participating in this study. All tests and procedures that will be done only for this study will be paid for by study funds. Eight thousand Tanzanian shillings (8000 Tshs), for transport costs and lunch, will be given to the participants each time they come to the clinics for follow up visits other than for routine care.

CONFIDENTIALITY

All the information that is obtained from you will be a secret and in addition to your hospital number, a study number will also be given. Only the investigator or somebody authorized by her will be able to link personal information back to study participants.

INCASE OF ANY QUESTION

If you have questions about the study or you want further information you should feel free to contact Tosi Michael Mwakyandile, the investigator, MUHAS, P.O. Box 65001, Dar es Salaam. Tel: +255-743 712 103

If you ever have questions about your rights as a participant, you may call Dr. Bruno Sunguya who is the chairman of MUHAS Senate Research and Publications Committee, P. O. Box 65001. Tel: 2150302 or the chairman of the National Health Research Ethics Committee (NatHREC), P.O.

Box 9653,Dar es salaam, Tanzania.Tel.: +255 22 2121400Mobile: +255 758 587885Hotline: +255 22 2130770Email: ethics@nimr.or.tz

Informed consent for participants:

I have read the information regarding the proposed study with the title above. I have also had an opportunity to discuss the study and ask questions to the investigators and I am satisfied that I understand what the study involves and my questions have been answered.

I agree (listed below) to take part in this study:

(1) ________________________________

Patient's Signature or thumb print _______________________ Date__________________

Witness' Signature (if patient cannot read) ________________________Date___________

6.1.2 Fomu ya Ridhaa

“FAIDA ZA DAWA YA ASPIRINI KWA MAENDELEO YA UGONJWA WA UKIMWI KWA WATU WALIOATHIRIKA NA VIRUSI VYA UKIMWI WANAOANZISHIWA DAWA ZA KUPUNGUZA MAKALI YA VIRUSI VYA UKIMWI.”

JINA LA MTAFITI: TOSI MICHAEL MWAKYANDILE

MFADHILI: HIS (HIV IMPLEMENTATION SCIENCE) AND THET

ANUANI: CHUO KIKUU CHA AFYA NA SAYANSI SHIRIKISHI CHA MUHIMBILI(MUHAS)

S. L. P 65001,

DAR-ES SALAAM.

Namba ya utambulisho: ________________________

MTAFITI

Utafiti pendekezwa wenye kichwa cha habari tajwa hapo juu utafanywana TOSI MICHAEL MWAKYANDILE, ambaye ni mwanafunzi wa shahada ya uzamivu katika idara ya famakolojia-MUHAS, chini ya usimamizi wa Prof. E. F. Lyamuya (MUHAS) na Dk. G**.** A. Shayo (MUHAS).

DHUMUNI LA UTAFITI

Maambukizi ya virusi vya ukimwi (VVU) bado ni tatizo la afya ya jamii haswa katika nchi za barani Afrika ikiwemo Tanzania. Dawa za kupunguza makali ya VVU zimeboresha idadi ya miaka ya kuishi ya watu wanaoishi na virusi vya UKIMWI ukilinganisha na wakati ambao zilikuwa bado hazijagunduliwa na kuanza kutumika. Kwa sababu hiyo, idadi ya matatizo ambayo kikawaida hayahusiani na VVU/UKIMWI kama vile saratani zisizo ambatana na UKIMWI, magonjwa ya ini, mapafu pamoja na moyo yameongezeka. Kuna ushahidi kuonyesha kwamba watu wanaoishi na VVU/ UKIMWI wana uwezekano Zaidi wakuumwa magonjwa ya moyo kuliko wale ambao hawaishi na VVU/ UKIMWI. Kuongezeka kwa ufanyaji kazi wa mojawapo ya aina ya seli katika damu zinazojulikana kama *pleteleti* na zile za mfumo wa kinga wa mwili unasemekana kuwa ni chanzo kikubwa kwenye ongezeko la uwezekano wa magonjwa ya moyo kwa watu wanaoishi na VVU/ UKIMWI. Matumizi ya dawa za kupunguza makali ya VVU huongeza idadi ya seli nyeupe za damu ambazo VVU hushambulia (CD4) pia hupunguza kiasi cha VVU katika damu. Zaidi ya hayo, pia idadi ya magonjwa nyemelezi hupungua. Hata hivyo matumizi ya dawa za kupunguza makali ya VVU hayakomeshi kabisa kuongezeka kwa ufanyaji kazi wa mfumo wa kinga wa mwili.

Kutokana na mapungufu haya ya dawa za kupunguza makali ya VVU juu ya kuongezeka kwa ufanyaji kazi wa mfumo wa kinga wa mwili, dawa ya ziada ni muhimu kwa ajili ya tiba nzuri ya maambukizi ya VVU.

Dawa ya aspirini (ASA), ni dawa inayodhibiti *inflamesheni* nakuzuia kuongezeka kwa ufanyaji kazi wa aina ya chembe hai ziitwazo *platelet.* Tafiti zilizofanyika zimeonyesha kuwa dawa hii hupunguza kuongezeka kwa ufanyaji kazi wa *pleteleti* na mfumo wa kinga ya mwili katika watu walioambukizwa VVU. Aidha, katika wagonjwa waliombukizwa VVU ASA imeonekana kuongeza idadi ya CD4 nakupunguza kiasi cha VVU. Hata hivyo, faida zote hizi zilionekana ndani ya muda mfupi na katika idadi ndogo ya washiriki. Utafiti huu unaopendekezwa ambao wewe unaalikwa kushiriki utaangalia faida za muda mrefu za ASA ikiwemo kupungua kulazwa hospitalini na/au kuhudhuria hospitalini mara kwa mara, vifo vinavyotokana na sababu yoyote pamoja na kasi ambayo UKIMWI huendelea kwenda hatua za juu. Inaaminika kuwa matokeo ya utafiti huu pendekezwa yatachangia kupungua kulazwa hospitalini na/ au kuhudhuria hospitalini mara kwa mara, vifo vinavyotokana na sababu yoyote na hivyo kuboresha hali na urefu wa maisha kwa washiriki wa utafiti na wagonjwa wengine wa siku za mbeleni.

Utafiti huu pia utaangalia athari za kuongeza dawa za ziada kwenye utumiaji wa dawa za kupunguza makali ya VVU.

WASHIRIKI WA UTAFITI

Wagonjwa watu wazima wa jinsia zote mbili ambao wamegundulika kuwa na vvu wakati huu au kabla lakini hawajawahi kutumia dawa za kupunguza makali ya vvu na wanatarajia kuanzishiwa hizo dawa, ambao wanahudhuria kliniki za matunzo na tiba kwa watu wenye maambukizi ya vvu zilizopo Mbagala Rangi Tatu na **Mwananyamala** wanaalikwa. Wale ambao wanapumu, wajawazito, wenye hatari ya kutokwa damu na/ au wale wanaotumia dawa zinazoathiri uwezo wa damu kuganda hawatoruhusiwa kushiriki. Wale wenye aleji (mzio)na ASA au bidhaa yoyote yenye ASA ndani yake pia hawatachukuliwa kwenye utafiti pendekezwa.

USHIRIKI UNAHUSISHA NINI?

Kama utakubali kushiriki katika utafiti huu, tutakuuliza maswali kuhusu afya yako na maisha yako; pia tutakupima urefu wako, uzito na shinikizo la damu. Sampuli za damu zitakuwa zinatolewa katika mahudhurio ya kliniki wakati waushiriki wako.

Utaratibu katika mahudhurio ya kliniki

Pindi utakapojiunga na utafiti huu utakuwa na mahudhurio **7** ya kliniki maalumu kwa utafiti huu; mwanzo kabisa utaangaliwa kama umekidhi vigezo vya ushiriki na utaandikishwa, halafu kutakuwa na mahudhurio ya kila mwezi kwa kipindi cha miezi sita. Baada ya kukubali kushiriki katika utafiti, vigezo vya ushiriki wako vitafanyiwa tathmini na kama umekidhi hivyo vigezo utachaguliwa kwa nasibu kuingia kwenye mojawapo ya makundi mawili ya dawa ambayo ni A na B. Hii itahakikisha kuwa kuingia kwako kwa kundi lolote la dawa ni kwa bahati pekee. Katika kipindi cha ufuatiliaji, washiriki kwenye makundi haya ya dawa watakuwa wakitumia dawa za kupunguza makali ya VVU lakini washiriki watakuwa pia wakitumia dawa linganishi isiyofanya kazi – *plasibo* katika kundi B na 75 mg ASA katika kundi A kutwa nyakati za jioni. Wakati ukiwa kwenye utafiti utahitajika kuja kliniki kila mwezi kwa ajili ya mahudhurio yaliyopangwa ya utafiti. Katika kila mahudhurio, utapewa dawa zako, maswali kuhusu afya yako yataulizwa, uzito wako utapimwa na sampuli za damu zitatolewa, katika baadhi ya mahudhurio, ilikuangalia maendeleo ya afya yako wakati wa tiba. Sampuli zitapelekwa kwenye maabara zilizopo MUHAS, MNH na Japan kwa ajili ya upimwaji. Utataarifiwa kuhusu majibu ya vipimo vyako vilivyopita vya maabara katika mahudhurio yanayofuata ya kila mwezi. Baada ya miezi sita ya utafiti, utaacha tiba na dawa za utafiti lakini utaendelea na kliniki yako na dawa zako za kupunguza makali ya VVU kama kawaida.

FAIDA ZA UTAFITI

Hakutakuwa na faida yoyote ya moja kwa moja au ya kifedha kwa ushiriki wako katika utafiti huu. Hata hivyo, ushiriki wako ni muhimu kwani utatusaidia kujua kama matumizi ya muda mrefu ya dawa ya ASA yataleta kupungua kwa kasi ya UKIMWI kuendelea kwenye hatua za juu, kupunguza kulazwa hospitalini na/ au mahudhurio hospitalini mara kwa mara, vifo vinavyotokana na sababu yoyote na hivyo kuboresha halina urefu wa maisha wa washiriki wautafiti na wagonjwa wa siku za mbeleni. Aidha, utafiti huu pendekezwa pia utasaidia kujua athari za kuongeza dawa za ziada kwenye utumiaji wa dawa za kupunguza makali ya VVU.

MADHARA YA KUSHIRIKI UTAFITI

Hatutegemei kuwepo kwa athari au madhara kwa mgonjwa kushiriki katika utafiti huu. Hata hivyo, kama ilivyo kwa dawa zote, kuna uwezekano wa athari ndogo sana ya madhara yanayohusishwa na dozi ndogo ya ASA ikilinganishwa na dozi kubwa. Hata hivyo, kama ilivyo kwa dawa yoyote,

kuna hatari ndogo kwamba unaweza ukapata athari mbaya zinazohusishwa na matumizi ya ASA. Kiasi kikubwa cha athari hizo sihatarishi, lakini tutakuwa tukifuatilia kwa karibu matukio hayo kwa ajili ya usalama wako na tutakuomba utoe taarifa ya dalili yoyote wakati wa mahudhurio ya kila mwezi au uwasiliane na timu ya watafiti.

Utajisikia maumivu kidogo kwa muda mfupi wakati wakutolewa damu ya uchunguzi, na inawezekana ukapata mchubuko kiasi au uvimbe ambao utatoweka ndani ya siku chache. Ujazo wa damu utakaotolewa ni mdogo (20 ml) na ni sawa na ule ambao kawaida unachukuliwa kama usingekuwa mshiriki wa utafiti huu na hautarajiwi kuleta madhara kwa afya yako.

UHURU WA KUSHIRIKI KATIKA UTAFITI

Tungependa kusisitiza kuwa kushiriki kwako kwenye utafiti huu ni hiari. Ukiamua kutoshiriki; haitaleta madhara kwenye tiba na huduma unayoipata kutoka hospitalini hapa. Vilevile uko huru kujitoa ushiriki wako wakati wowote ule wa utafiti. Kama utataka tuziharibu sampuli zako za vipimo tutafanya hivyo. Uamuzi wako wowote wa aina hiyo utaheshimiwa na hautaathiri hali ya huduma ya afya ambayo utaipata hapa katika kliniki za matunzo na tiba kwa watu wenye maambukizi ya VVU au kwingineko.

MALIPO YA KUSHIRIKI KATIKA UTAFITI.

Hakutakuwa na malipo ya aina yoyote ile kwa kushiriki kwenye utafiti huu. Vipimo vyote vitakavyofanyika kwa ajili ya utafiti tu vitalipiwa kwa gharama ya utafiti huu. Shilingi elfu nane za kitanzania (8000 Tshs), kwa ajili ya nauli na chakula cha mchana, zitalipwa kwa kila mshiriki kila mara atakapokuja kliniki nje ya ratiba zao za kawaida.

USIRI

Taarifa zote zitakazopatikana kutoka kwako zitakuwa ni za siri, ukiacha namba yako ya hospitali utapewa namba nyingine ya utafiti huu. Ni mtafiti tu na mtu mwingine atakayeruhusiwa kisheria na mtafiti watakuwa na uwezo wa kuhusisha taarifa binafsi za washiriki wa utafiti.

KWA MASWALI YOYOTE

Kama una maswali kuhusu utafiti au unataka taarifa Zaidi unapaswa kujisikia huru kuwasiliana na Dk. Tosi Michael Mwakyandile, ambaye ndiye Mtafiti Mkuu, MUHAS, S. L. P. 65001, Dar es Salaam. Namba ya simu: +255-743 712 103

Kama ukiwa na maswali kuhusu haki zako kama mshiriki, unaweza kumpigia simu Dk. Bruno Sunguya ambaye ni Mwenyekiti wa Kamati inayosimamia maadili ya tafiti ya Seneti ya MUHAS, S. L. P. 65001. Simu: 2150302 au mwenyekiti wa kamati ya taifa inayosimamia maadili ya utafiti wa afya (NatHREC), S. L. P. 9653, Dar es salaam, Tanzania. Simu: +255 22 2121400 Simu ya mkononi: +255 758 587885 Simu ya masaa ishirini na nne: +255 22 2130770 barua pepe: ethics@nimr.or.tz.

.

Ridhaa kwa ajili ya washiriki:

Nimesoma habari kuhusu utafiti huu. Mimi pia nilikuwa na nafasi ya kujadili utafiti na kuuliza maswali kwa watafiti na nimeridhika kwamba nimeelewa nini utafiti unahusisha na maswali yangu yamejibiwa.

Mimi nakubali (hapa chini) kushiriki katika utafiti huu:

(1) ________________________________

mshiriki

sahihi au dole gumba_______________________ Tarehe__________________

shahidi

Sahihi (kama mshiriki hawezi kusoma) ________________________ Tarehe___________

Jina la shahidi: ____

Ninathibitisha kwamba maelezo ya juu nilielezwa kwa maneno na kwamba yeye anaelewa asili na madhumuni ya utafiti na mimi nakubaliana na ushiriki katika utafiti wa mgonjwa hapo juu.

Mimi nimempa nafasi ya kuuliza maswali ambayo yamejibiwa na kuridhisha.

Afisa Utafiti

sahihi _____________________________________ Tarehe__________________

Jina la afisa utafiti: ____

6.2 Appendix 2 – Case Report Form for Visit 0

Project; The effect of aspirin on human immunodeficiency virus (hiv) disease progression among hiv- infected individuals initiating anti- retroviral therapy.

PARTICIPANT ELIGIBILITY FORM (*To be filled in on* ***Visit 0****)*

Visit date_ _/ _ _/ _ _ _ _

Site

Mbagala Rangi Tatu	
**Mwananyamala**	

Informed consent

YES		
NO		Reason;

I: Basic and Social Demographic Information

1. Patient's Initials_ _ _
2. Screening Number_ _ _ _ _
3. Date of Birth_ _/ _ _/ _ _ _ _
4. Age_ _ _ (years)
5. Sex:

Male	
Female	

6. Residence:

Kinondoni	
Temeke	
Ilala	
Kigamboni	
Ubungo	
Out of Dar es Salaam	

7. Level of Education:

No formal education	
Primary education	
Secondary education	
Higher education	
Other(specify)	

8. Employment Status:

Unemployed	
Employed	
Self- employed	

9. Marital Status:

Single	
Married	
Cohabiting	
Divorced	
Widow	
Widower	

II: Medical History

i. History of other medical conditions

Is the participant asthmatic?		
Does the participant have any bleeding diatheses?		
Does the participant have active or history of peptic ulceration or gastrointestinal bleeding within the last year?		

ii. Drug history (for both prescription and over the counter medications)

Does the participant have history of intolerance or allergy to ASA or any ASA containing products or NSAIDs or celecoxib or other salicylates or sulphonamides?		
Is the participant on antithrombotic/ antiplatelet therapy?		
Is the participant on current or previous regular use of ASA at any dose or current use of another NSAID?		
Is the participant on current or long term use of oral corticosteroids?		

III: Screening Investigations:

1. Was UPT done? 1. YES____ Results: _____________ (Positive/Negative/Not Applicable)
   2. No_____Reason____

2. Was serum creatinine done? 1. YES________Results: Serum creatinine_________ mg/dL
   2. No____Reason_____________________________________

IV: Inclusion criteria

*All answers to questions 1-6 must be **YES** for the subject to be eligible.*

Serial No	QUESTION	RESPONSE	
		YES	NO
1.	Has the participant given an informed consent?		
2.	Is the participant HIV- infected?		
3.	Is the participant ARV drugs naïve?		
4.	Is the participant 18 years old or above?		
5.	Is the participant attending CTC at MbagalaRangiTatu or **Mwananyamala**		
6.	Is the participant residing in Dar es Salaam?		

V: Exclusion criteria

*All answers to questions 1 -9 must be **NO** for the subject to be eligible.*

Serial No	QUESTION	RESPONSE	
		YES	NO
1.	Does the participant have history of intolerance or allergy to ASA or any ASA containing products or NSAIDs or celecoxib or other salicylates or sulphonamides?		
2.	Is the participant asthmatic?		
3.	Does the participant have any bleeding diatheses?		
4.	Does the participant have active or history of peptic ulceration or gastrointestinal bleeding within the last year?		
5.	Is the participant on antithrombotic/ antiplatelet therapy?		
6.	Is the participant on current or previous regular use of ASA at any dose or current use of another NSAID?		
7.	Is the participant on current or long term use of oral corticosteroids?		
8.	Is the participant's urine pregnancy test positive?		
9.	Does the participant have history of moderate or severe renal impairment, with eGFR < 45 ml/ min/ 1.73 m^2		

VI: SELECTION OF PARTICIPANTS INTO THE TRIAL

1. Is the participant eligible? ____(YES/NO) If no why? ______________________________
2. Is the participant selected into the study? _____(YES/NO) If no why? ___________________________________
3. Participant's study number_ _ _ _ _
4. Investigator's signature_____________________Date_ _/_ _/_ _ _ _

BASELINE INFORMATION FORM AFTER SELECTION INTO THE STUDY (*To be filled in on* ***Visit 0****)*

A. MEDICAL HISTORY

i. HIV/AIDS specific history

1. Date of first diagnosis with HIV- infection_ _/ _ _/ _ _ _ _
2. Date of ART initiation_ _/_ _/_ _ _ _
3. ART regimen initiated ____

ii. Smoking status

1. Current		2. Past		3. Never	
For how long have you been smoking?...........years					
How many cigarettes per day?					

iii. Alcohol drinking habit

1. Yes		2. No	
What type of alcohol do/did you take?			
How many bottles per day?			

iv. Do you use substance of abuse?

1. Yes		2. No	
What type of substance of abuse do/did you take?			
For how long have you been using/ did you use the substance of abuse?			
How often do/ did you take?			

v. Other known chronic diseases:

		YES	How long(months)
1.	Hypertension		
2.	Diabetes		
3.	Others (specify)		

B. HISTORY OF CONCOMITANT MEDICATIONS:

Provide the following information for all medications including over the counter medications.

Serial Number	Medication Name	Daily dose	Reason for Use	Start Date MM/DD/YY	Stop Date MM/DD/YY

C. PHYSICAL EXAMINATION

A. VITAL SIGNS and ANTHROPOMETRIC MEASUREMENTS

Temperature ___. ___ºC

Respiratory rate ____ per min

Pulse____bpm

Blood pressure

	Measurement 1	Measurement 2	Average measurement
Systolic (mm Hg)			
Diastolic (mm Hg)			

Weight____. ____kg

Height____. _____m

BMI= ______. ___kg/m^2

BMI category ___________________

B. SYSTEMIC EXAMINATION

Body system	Normal	Abnormal	Not done	Comments
General				
Skin and mucous membranes				
Lymphatic				
Respiratory				
Cardiovascular				
Gastrointestinal tract				
Central nervous system				
Musculoskeletal				

C. CLINICAL LABORATORY DATA

Was sample for haematology taken? _____(YES/NO) If No why? ____

Test		Results	Units	Comment
HAEMAT	Haemoglobin			
	Haematocrit			
	RBC			

	WBC			
	Neutrophils			
	Lymphocytes			
	Monocytes			
	Bands			
	Eosinophils			
	Basophils			
	Other			
	Platelet Count			

Was sample for lipid profile taken? _____(YES/NO) If No why? ___________________________________

**Test**		**Results**	**Units**	**Comment**
LIPID PROFILE	Cholesterol			
	Triglycerides			
	HDL			
	LDL			

Was sample for liver function tests taken? _____(YES/NO) If No why? ___________________________________

		**YES**	**NO**			
LIVER FUNCTION TESTS	Total bilirubin					
	AST					
	ALT					

Was sample for renal function tests taken? _____(YES/NO) If No why? ________________________________

Test		Results	Units	Comment
RENAL FUNCTION TESTS	BUN			
	Creatinine			

Was sample for biomarkers taken? _____(YES/NO) If No why? ________________________________

Test		Results	Units	Comment
Biomarkers	sCD14			
	sP- selectin			
	Viral load			
	CD4 count			
	CD38 and HLA-DR			
	PD-1			

D. STUDY DRUG DISPENSING

Thirty-day supply

Drug	Strength per pill	Number of pills given
ARV drugs		

Thirty- day supply

Drug	Strength per pill	Number of pills given
ASA/ Placebo		

6.3 Appendix 3 – Case Report Form for Subsequent visits

Participant study number _ _ _ _ _

Participant initials _ _ _

Visit number _ _

Visit date _ _/ _ _/ _ _ _ _

A. MEDICAL HISTORY

1. ART regimen change? ___ (YES/ NO) if yes why? ____
2. Date of regimen change_ _/ _ _/ _ _ _ _
3. New ART regimen initiated ____________________________________

i. Smoking status (please check from previous visit if stopped/ started/ continuing)

1. Current		2. Past		3. Never	

ii. Alcohol drinking habit (please check from previous visit if stopped/ started/ continuing)

1. Yes		2. No	

iii. Do you use substance of abuse? (please check from previous visit if stopped/ started/ continuing)

1. Yes		2. No	

iv. HISTORY OF CONCOMITANT MEDICATIONS: (please check from previous visit if stopped/ started/ continuing)

Provide the following information for all medications including over the counter medications.

Serial Number	Medication Name	Daily dose	Reason for Use	Start Date MM/DD/YY	Stop Date MM/DD/YY	If continuing

B. PHYSICAL EXAMINATION

i. VITAL SIGNS and ANTHROPOMETRIC MEASUREMENTS

Temperature ___. ___ºC

Respiratory rate ____ per min

Pulse____bpm

Blood pressure

	Measurement 1	Measurement 2	Average measurement
Systolic (mm Hg)			
Diastolic (mm Hg)			

Weight____. ____kg

Height____. _____m (use baseline)

BMI= ______. ___kg/m^2

BMI category __________________

ii. SYSTEMIC EXAMINATION

**Body system**	**Normal**	**Abnormal**	**Not done**	**Comments**
General				
Skin and mucous membranes				
Lymphatic				
Respiratory				
Cardiovascular				

Gastrointestinal tract				
Central nervous system				
Musculoskeletal				

C. <u>CLINICAL LABORATORY DATA</u>

Was sample for haematology taken? _____(YES/NO) If No why? ___________________________________

Test		Results	Units	Comment
HAEMATOLOGY	Haemoglobin			
	Haematocrit			
	RBC			
	WBC			
	Neutrophils			
	Lymphocytes			
	Monocytes			
	Bands			
	Eosinophils			
	Basophils			
	Other			
	Platelet Count			

Was sample for lipid profile taken? _____(YES/NO) If No why? ___________________________________

Test		Results	Units	Comment
LIPID PROFILE	Cholesterol			
	Triglycerides			
	HDL			
	LDL			

Was sample for liver function tests taken? _____(YES/NO) If No why? ___________________________________

Test		Results	Units	Comment
LIVER FUNCTION TESTS	Total bilirubin			
	AST			
	ALT			

Was sample for renal function tests taken? _____(YES/NO) If No why? ___________________________________

Test		Results	Units	Comment
RENAL FUNCTION TESTS	BUN			
	Creatinine			

Was sample for biomarkers taken? _____(YES/NO) If No why? ___________________________________

Test		Results	Units	Comment
Biomarkers	sCD14			
	sP- selectin			
	Viral load			
	CD4 count			
	CD38 and HLA-DR			
	PD-1			

D. COMPLIANCE TO STUDY MEDICATION

Number of pills returned: _____

Start date of drug for this period: _ _/_ _/_ _ _ _

Stop date of drug for this period: _ _/_ _/_ _ _ _

Number of pills that should have been taken	Number of Pills remaining	Number of pills actually taken	% of Pills taken
**Comments**			

E. ADHERENCE TO ART

Number of pills returned: _____

Start date of drug for this period: _ _/_ _/_ _ _ _

Stop date of drug for this period: _ _/_ _/_ _ _ _

Number of pills that should have been taken	Number of Pills remaining	Number of pills actually taken	% of Pills taken
**Comments**			

F. STUDY DRUG DISPENSING

Thirty-day supply

Drug	Strength per pill	Number of pills given
ARV drugs		

Thirty- day supply

Drug	Strength per pill	Number of pills given
ASA/ Placebo		

G. ADVERSE EVENTS

At the end of study check if none: ____

**Adverse event**	**Start Date (MM/ DD/YY)**	**Stop Date (MM/ DD/YY)**	**Event Recovery Status(Resolved or Not Resolved)**	**Check if Continuing**	**Relationship to study drug**	**Toxicity Grade**	**Seriousness**

H. MORBIDITY

Start date of assessment for this period: _ _/_ _/_ _ _ _

Stop date of assessment for this period: _ _/_ _/_ _ _ _

	Number of hospital/ health facility visits	Number of hospital/ health facility admissions
Reasons for visit or admission respectively		

I. OFF STUDY FORM

Date on Follow- up _ _/_ _/_ _ _ _

Date off Follow- up _ _/_ _/_ _ _ _

Date off study _ _/_ _/_ _ _ _

Date of Last Contact _ _/_ _/_ _ _ _

Date Last Study Medication Taken _ _/_ _/_ _ _ _

Reason Off Study

Completed Study	
Adverse Event	
Lost to Follow- up	
Death	
Other(please specify in Comments section)	
Comments	

Continuing Adverse Event

Adverse Event: ____

Start Date of Event: _ _/_ _/_ _ _ _

Outcome: ____________________

6.4 Appendix 4 – Death Report Form

(Please complete this form only when death of the participant is reported)

Participant study number _ _ _ _ _

Date of completion of the form _ _/ _ _/ _ _ _ _

Date of Death_ _/ _ _/ _ _ _ _

Place of death	1. Hospital		Other(specify)	
Autopsy performed?	YES		NO	
Cause of death/ condition associated with death	Study treatment		Other, please specify	
Comments				

6.5 Appendix 5 – Prohibited medications during the study (also used for eligibility check)

**Anti-coagulants:**	Warfarin	**Anti-platelets:**	Clopidogrel
	Acenocoumarol		Dipyridamole
	Phenindione		Prasugrel
	Dabigatran		Ticagrelor
	Unfractionated Heparin		Abciximab
	Low molecular weight heparin(1)		Tirofiban
	Rivaroxaban		Eptifibatide
	Apixaban		Epoprostenol
	Argatroban		Fondaparinaux

**LONG-TERM NSAIDS(2)**	Ibuprofen	**Aspirin:** (including over the counter)	Nu-seals
	Naproxen		Anadin
	Diclofenac		Beechams powders
	Acelofenac		Alka-seltzer
	Fenoprofen		Disprin
	Flurbiprofen		Codis 500
	Ketoprofen		
	Dexketoprofen		
	Tiaprofenic acid	**Others:**	
	Etodolac		Methotrexate
	Indomethacin		Long-term corticosteroids (3)
	Meloxicam		(e.g. dexamethasone,
	Tenoxicam		
	Nabumetone		
	Phenylbutazone		

Ketorolac<br>Piroxican<br>Sulindac<br>Tolfenamic acid<br>Celecoxib<br>Etoricoxib	prednisolone,<br>hydrocortisone)

1. Low molecular weight heparin at a prophylactic dose for inpatient thromboembolism is permitted.

2. Non-steroidal anti-inflammatory drugs (NSAIDs) should be avoided wherever possible but short term intermittent NSAID use is allowed. NSAIDs should not be co-administered with the trial treatment for more than 2 consecutive weeks). Paracetamol can be considered as an alternative analgesic and is permitted within the trial.

3. Short term intermittent systemic corticosteroids are permitted however longer term use (longer than 2 continuous weeks) is not permitted.

6.6 Appendix 6 – Definitions of some terms

1. Regular use of ASA is defined as taking ASA (at any dose) more than twice in any given week for more than 4 consecutive weeks.
2. Current NSAID use is defined as taking any NSAID for more than a week in the preceding month.

6.7 Appendix 7 – Formula for calculating eGFR

eGFR= 175 x $(S_{Cr})^{-1.154}$ x $(age)^{-0.203}$ x 0.742 [if female] x 1.212 [if Black]

Abbreviations / Units

eGFR (estimated glomerular filtration rate) = mL/min/1.73 m^2

S_{cr} (standardized serum creatinine) = mg/dL

age = years

6.8 Appendix 8 – Bleeding disorders

Type	Underlying pathology	Bleeding disorder
Platelet count	Thrombocytopenia	-Thrombocytopenic purpura:Immune thrombocytopenia (Evans syndrome)<br>Thrombotic. Microangiopathy<br>(Thrombotic Thrombocytopenic purpura,<br>Upshaw Schulman syndrome)<br>-Heparin-induced thrombocytopenia<br>-May–Hegglin anomaly
Platelet function	Adhesion	Bernard–Soulier syndrome
	Aggregation	Glanzmann's thrombasthenia
	Platelet storage pool deficiency	Hermansky–Pudlak syndrome,<br>Gray platelet syndrome
Clotting factor		Hemophilia A/VIII, B/IX, C/XIVon Willebrand disease<br>Hypoprothrombinemia/II<br>Factor VII deficiency<br>Factor X deficiency<br>Factor XII deficiency<br>Factor XIII deficiency<br>Dysfibrinogenemia<br>Congenital afibrinogenemia

6.9 Appendix 9 – Casuality and assessment of severity- Adverse Events

Adverse events include:

- An exacerbation of a pre-existing illness
- An increase in frequency or intensity of a pre-existing episodic event or condition
- A condition (even though it may have been present prior to the start of the trial) detected after trial drug administration
- Continuous persistent disease or a symptom present at baseline that worsens following administration of the study treatment
- Any clinically relevant deterioration in any laboratory assessments or clinical tests

Adverse events will not include:

- A pre-existing condition (unless it worsens significantly during treatment).
- Diagnostic and therapeutic procedures, such as surgery (although the medical condition for which the procedure was performed must be reported if new)

The severity of an Adverse Event will be assessed as follows:

- **Mild**: Events that require minimal or no treatment and do not interfere with the patient's daily activities.
- **Moderate**: Events that cause sufficient discomfort to interfere with daily activity and/or require a simple dose of medication.
- **Severe**: Events that prevent usual daily activity or require complex treatment.

The relationship of the event to the study drug will be assessed as follows:

RELATIONSHIP	DESCRIPTION	SAE TYPE
**1. Unrelated**	There is no evidence of any causal relationship.	**Unrelated SAE**
**2. Unlikely**	There is little evidence to suggest that there is a causal relationship (for example, the event did not occur within a reasonable time after administration of the trial medication). There is another reasonable explanation for the event (for example, the participant's clinical condition, other concomitant treatment).	**Unrelated SAE**
**3. Possible**	There is some evidence to suggest a causal relationship (for example, because the event occurs within a	**SA reaction**

	reasonable time after administration of the trial medication). However, the influence of other factors may have contributed to the event (for example, the participant's clinical condition, other concomitant treatments).	
**4. Probable**	There is evidence to suggest a causal relationship and the influence of other factors is unlikely.	**SA reaction**
**5. Definitely**	There is clear evidence to suggest a causal relationship and other possible contributing factors can be ruled out.	**SA reaction**

6.10. Appendix 10 – Procedure for PBMC separation

Procedure for isolation of mononuclear cells

1. Add 5mil of ficoll in each 15ml tube
2. 2x volumes of RPMI only added to cellular sediments.
3. Carefully add the diluted blood sample (10ml) onto the Ficoll-Paque media solution. Important: When layering the sample do not mix the Ficoll-Paque media solution and the diluted blood sample.
4. Centrifuge at 2000rpm for 20 min at 18ºC to 20°C (brake should be turned off).
5. Prepare two 50ml tubes. Add 15ml of RPMI into each (3X volume of mononuclear layer)
6. Draw off the upper layer containing mononuclear cells.
7. Transfer the layer of mononuclear cells to a sterile 50ml centrifuge tube containing 15ml of RPMI only using a sterile pipette.

Washing the cell isolate

8. Centrifuge at 1500rpm, 20ºC fast
9. Discard supernatant
10. Resuspend cells in 2 ml of FCS containing 10% DSMO
11. Store in cryotubes 1 ml for each sample at -70ºC
