# Supplementary material for: Low-dose aspirin is not effective as an adjunct treatment for HIV infection among people living with HIV on dolutegravir-based antiretroviral therapy: A randomised double-blind, parallel-group placebo-controlled trial
Source: PLoS One. 2025 Aug 29;20(8):e0331087. doi: 10.1371/journal.pone.0331087 (PMC12396663; doi:10.1371/journal.pone.0331087)
Supplement: Attachment — Submitted filename: Responses to reviewers .docx [file pone.0331087.s016.docx]

**Responses to points raised by the academic editor and reviewers on a manuscript titled ‘Low-dose aspirin is not effective as an adjunct treatment for HIV infection among people living with HIV on dolutegravir-based antiretroviral therapy: a randomised double-blind, parallel-group placebo-controlled trial.’**

**Manuscript ID**- PONE-D-25-05215.

| **Reviewer** | **Reviewers’ Comments** | **P.I Responses** | **Changes inserted in Text on Page and Line Nos in the file labeled ‘Manuscript’.** |
| --- | --- | --- | --- |
| **Journal requirements** | When submitting your revision, we need you to address these additional requirements.   1. Please ensure that your manuscript meets PLOS ONE's style requirements, including those for file naming. The PLOS ONE style templates can be found at   <https://journals.plos.org/plosone/s/file?id=wjVg/PLOSOne_formatting_sample_main_body.pdf>  and  <https://journals.plos.org/plosone/s/file?id=ba62/PLOSOne_formatting_sample_title_authors_affiliations.pdf> | Comment appreciated. The manuscript has been revised to meet PLOS ONE’s style requirements, including those for file naming as per the two links shared in the comment. The revisions made are: | Throughout the manuscript and Supporting Information |
|  |  | Removing abbreviation of affiliation and listing it in full | Page 1(Lines 9,11 and 17) |
|  |  | Writing headings in sentence case | Page 2 (Line 23),  Page 3 (Line 49),  Page 5 (Line 93),  Page 7 (Line 153),  Page 8 (Line 174),  Page 12 (Line 270),  Page 15 (Line 338),  Page 22 (Line 425) |
|  |  | Use of level 1 heading, bold type 18pt font for all major sections | Page 2 (Line 23),  Page 5 (Line 93),  Page 7 (Line 153),  Page 15 (Line 338),  Page 22 (Line 425),  Page 24 (Line 484),  Page 25 (Lines 491, 499),  Page 31, Line 501 |
|  |  | Use of level 2 heading, bold type 16pt font for sub-sections of major sections. | Page 7 (Line 155,),  Page 8 (Lines 165, 174, 184),  Page 9 (Line 207),  Page 10 (Line 227),  Page 12 (Line 270),  Page 13 (Line 286),  Page 14 (Line 315),  Page 15 (Lines 333, 349),  Page 17 (Lines 359, 367),  Page 20 (Lines 386, 395) |
|  |  | Use of level 3 heading, bold type 14pt font for sub-sections of major sections. | Page 10 (Line 252),  Page 11, (Line 262) |
|  |  | Removing italics where not needed | Page 2 (Lines 25, 29, 42, 43, 46),  Page 3 (Line 47),  Page 14 (Line 313),  Page 15 (Lines 345, 347, 353),  Page 17 (Line 363, 371, 373), Table 2  Page 18 (Lines 375, 376, 378),  Page 19 (Table 3),  Page 20 (Lines 392, 403, 405),  Page 21 (Line 420),  Page 22 (Line 422),  Supporting Information Tables |
|  |  | Removing bold texts where not needed | Page 2 (Line 43),  Page 17 (Table 2),  Supporting Information Tables |
|  |  | Using bold type for titles of Tables and Figures | Page 15 (Line 348 for Fig 1),  Page 16 (Lines 356-357, for Table 1),  Page 17 (Line 365, for Table 2),  Page 19 (Lines 382-383, for Table 3),  Page 20 (Lines 393-394, for Fig 2),  Page 21 (for Table 4)  Page 22 (Line 423, for Fig 3) |
|  |  | Including Table directly after the paragraph in which they are first cited. | Page 21 (for Table 4) |
|  |  | Uploading supporting information separately as individual files | - |
|  |  | Formatting references per required style by PLoS One. | Reference section  Page 25 (References 1, 4-5),  Page 26 (References 6, 8-10, 13),  Page 27 (References 16-18, 21),  Page 28 (References 22-23, 26-28),  Page 29 (References 29-32, 34,35),  Page 30 (References 38, 40, 42),  Page 31 (References 43-45) |
|  |  | Editing Supporting Information Captions at the end of the manuscript | Pages 31-33 |
|  |  | Naming of Supporting Information File | All Supporting Information Files, |
|  | 2. We note that the original protocol that you have uploaded as a Supporting Information file contains an institutional logo. As this logo is likely copyrighted, we ask that you please remove it from this file and upload an updated version upon resubmission. | Comment appreciated. The institutional logo in the Supporting Information file has been removed and an updated version without the logo has been uploaded. | S3_File.pdf |
|  | If the reviewer comments include a recommendation to cite specific previously published works, please review and evaluate these publications to determine whether they are relevant and should be cited. There is no requirement to cite these works unless the editor has indicated otherwise. | Comment noted however, this is not applicable for our manuscript. The reviewer comments did not include a recommendation to cite specific previously published works. | - |
|  | Please review your reference list to ensure that it is complete and correct. If you have cited papers that have been retracted, please include the rationale for doing so in the manuscript text, or remove these references and replace them with relevant current references. Any changes to the reference list should be mentioned in the rebuttal letter that accompanies your revised manuscript. If you need to cite a retracted article, indicate the article’s retracted status in the References list and also include a citation and full reference for the retraction notice. | Comment noted. The reference list has been revised and now all the references are written as per style required by PLoS One. No reference has been added or removed. | Reference section  Page 25 (References 1, 4-5),  Page 26 (References 6, 8-10, 13),  Page 27 (References 16-18, 21),  Page 28 (References 22-23, 26-28),  Page 29 (References 29-32, 34,35),  Page 30 (References 38, 40, 42),  Page 31 (References 43-45) |
| **Reviewer: 1** | The design of the study was a simple randomized parallel strategy with adequate sample size and power utilized. The data analysis was routine for the objectives. A logistic regression model using generalized estimating equations (GEE) was applied to assess the differences between the treatment arms in proportions of participants with virologic suppression or failure at baseline and 24 weeks. McNemar test was used to compare the proportions of participants in the treatment arms with HVL < 50 and HVL ≥ 1,000 RNA copies/mL at baseline and 24 weeks. A Linear regression model using GEE was used to analyze differences in CD4 count, markers of platelet activation, immune activation, and exhaustion between the arms at baseline, 12 and 24 weeks. In both types of analyses, the interaction of time and treatment arm represented the treatment effect.  The overall conclusion that Low-dose aspirin initiated with ART through 24 weeks did not impact virologic or immunologic markers among PLHIV was supported by the analysis performed. On a minor note, the detail results of the logistic regression could have been enhanced. What exactly were the variables considered in the model and were there any statistically significant confounders or possible confounders? One suspects not. However, some clarification would help. | Comment noted with thanks. In the logistic regression model, since this was a randomised study, we had treatment arm as the only predictor for virologic outcome. A statement to reflect this has been added in the study sample size and statistical analysis section under materials and methods to clarify. | Page 13 (Lines 299-300) |
|  | Also, was a visit or admission to a health facility for medical attention/care due to illness the only morbidity? | Comment noted. In this study, morbidity was assessed by asking the participants if they had visited or were admitted to a health facility for medical attention/care due to an illness since the last visit. A positive response qualified morbidity, however the description of the exact diagnosis or condition were not captured due to the complexity of tracing clinical records from facilities where participants attended. Any other complaints participants had without visiting health facility were considered adverse events. | - |
|  | The authors have , to their credit, noted the high lost to follow up as a study limitation. Perhaps future strategies should focus on recruitment retention given the short duration of the study. | Comment appreciated. We agreed with Reviewer 1 that in recruitment retention strategies need to be planned and implemented especially for short follow up clinical trials. However, the emergency of the COVID-19 pandemic during theisstudy may have contributed to high lost to follow up observed. | - |
| **Reviewer: 2** | Overall, the study has a robust design with clearly defined outcomes and transparency. The attrition seems to be balanced in both cohorts. Although all time points for primary outcome could not be achieved, 24-month analysis for primary outcome is acceptable in view of longitudinal nature of this disease, as well as employed equivalent duration of aspirin treatment. Apart from minor corrections in verbiage that authors should re-read and make, I do not have notable comments. | Comment appreciated. We have re-read and revised the manuscript to correct the minor verbiage issues by removing wordiness and unnecessary modifiers and using consistent terminologies | Throughout manuscript  Page 5 (Lines 96,97, 100),  Page 6 (Lines 117, 120, 124, 125, 127, 129, 130, 132),  Page 8 (Lines 176, 181, 183),  Page 10 (Lines 212, 220-226),  Page 15 (Line 344),  Page 22 (Lines 437, 440) |
